# To pool or not to pool? Trends and predictors of banking arrangements within Australian couples

**DOI:** 10.1371/journal.pone.0214019

**Published:** 2019-04-17

**Authors:** Yangtao Huang, Francisco Perales, Mark Western

**Affiliations:** ARC Centre of Excellence for Children and Families over the Life Course, Institute for Social Science Research, The University of Queensland, Brisbane, Australia; University of Jyvaskyla, FINLAND

## Abstract

The study of domestic money goes at the heart of debates about independence and equality in intimate relationships. It provides an important window on the individualization of family life and how couples reconcile ideals around egalitarian marriage ideologies with enduring gender inequality in society and the labor market. This study approaches these issues from the prism of couples’ banking arrangements (separate vs. joint accounts), an aspect of financial organization that approximates the executive management of household resources and which has received comparatively little attention. As such, it is amongst the first to deploy large-scale, household panel data (*Household*, *Income and Labour Dynamics in Australia Survey*, *n* = 15,379 observations from 7,054 couples) and binary and multinomial random-effect logistic regression models to examine trends over time in couples’ banking arrangements and their socio-demographic predictors. Key findings indicate that a large share of couples in Australia favors ‘mixed’ bank account strategies (i.e., holding both joint and separate accounts), but ‘egalitarian’ choices (i.e., dual separate accounts) are prevalent and on the rise. Couples’ bank account choices are influenced in theoretically-meaningful ways by economic resources, transaction costs, relationship history, gender-role attitudes, and family background.

## 1 Background

Despite the significance of how personal resources are integrated into the family unit for the study of marriage and cohabitation [1–3], the ways in which money, power and control are negotiated within couple relationships remained a ‘black box’ for family researchers until the mid 20^th^ century [4–5]. This paucity of research stemmed partially from the fact that money was—and is—an intensely private matter, rarely discussed outside of the couple dyad [2,6,7]. As a result, early studies on the inner financial workings of families accepted largely untested assumptions, including that there was little variation across families in financial management practices, or that resources were shared equally amongst family members [4]. Since the 1980s, sociological work made influential theoretical and qualitative contributions that set up the building blocks for subsequent inquiry into the ways in which couples organize their money. As noted by key authors, an important aspect of the household financial organization is whether partners pool their money in a joint bank account, manage it autonomously in separate accounts, or a mixture of both [2,4,7–9]. Understanding how couples manage their money is an important endeavor, as couples’ banking practices offer unique insights into the individualization of marriage and family life [10–12] and how individuals reconcile ideals around marriage as an egalitarian relationship with prevailing gender inequalities in society and the labor market [1–2]. Within-couple financial arrangements have also been linked to a host of individual and family outcomes [13–15]. All in all, the study of domestic money goes at the heart of debates about independence and equality in intimate relationships [2].

Despite the significant implications of how couples manage their day-to-day finances and the fact that all couples must confront these decisions, quantitative research into these issues has not developed at the same pace as qualitative and theoretical research [13,14,16]. In fact, only a handful of studies using robust statistical methods, representative samples and/or longitudinal data have examined the precursors and consequences of couples’ banking arrangements [1,7,14,17]. This is limiting, as quantitative inquiry into couples’ banking arrangements can help prove or disprove long-standing theoretical propositions, generalize findings from small-scale qualitative projects, and quantify relationships between socio-cultural factors, decision-making processes and couples’ banking arrangements. The present study is one of the first to use national longitudinal survey data to examine trends over time in couples’ banking arrangements and their socio-demographic predictors. In doing so, it expands upon earlier studies of within-couple banking arrangements in several ways.

First, we provide a more systematic examination of couples’ banking arrangements by comparing the predictive power of a wider set of theoretically-relevant factors, including economic factors (absolute and relative income, number of dependent children), life-course factors (relationship history and duration), socio-cultural factors (gender-role attitudes), and intergenerational effects (socio-economic background, maternal empowerment).

Second, we innovate methodologically by deploying longitudinal survey data (to examine change over time) and panel regression models (to improve estimation by accounting for additional sources of confounding), and by using information from both couple members (which reduces measurement error due to misreporting).

Third, we provide one of the first robust quantitative accounts of couples’ banking arrangements in Australia. Australia is an interesting case study due to its institutional legacy, and so our study contributes to enriching cross-national comparisons. In particular, for most of the 20^th^ century Australia had a state-instituted pay setting system consisting of a regulated “family wage” for male jobs, and half the male earning rate for women in the same jobs. These practices deterred women’s labor force participation and institutionalized a male breadwinner model in which men were considered to be the ‘financial leaders’ within their households [18]. A legacy of these institutional arrangements is the historical and contemporary high prevalence of female part-time work in Australia, which entrenches women’s financial dependence on their male partners [19]. To the extent that these factors have remained embedded in the Australian social ethos, the banking arrangements of couples in Australia may exhibit different patterns than those in other developed countries–such as the US, Norway or the UK. Studying intra-household money management in countries with diverging institutional profiles is important [16] and, as Pahl puts it, *“assumptions about family finances developed in Europe and North America may not apply in other parts of the world”* [20].

## 2 Conceptual framework

In this section we derive a conceptual framework on within-couple financial arrangements to guide our empirical analyses. We also discuss the available empirical literature, prior to developing testable research hypotheses.

### 2.1 Individualization, modernization and the joint account

Pahl and Vogler were amongst the first to document how, by the 1980s, already a majority of couples pooled their finances in joint accounts [8,21–23]. This finding was echoed in concomitant and subsequent studies in the US, Norway, Sweden, New Zealand or Australia [1,2,7,24–26]. Today, joint accounts are held in a majority of couple relationships in developed countries [4,13]. Despite the present-day ubiquity of the joint account, some have documented shifts towards financial separateness in recent years [4,12–14,27]. Changes in how couples handle money are embedded within wider processes of individualization and modernization of marriage and personal relationships [5], and have been linked to discourses about marriage as an institution moving towards the notion of ‘companionate marriage’ [12]. For instance, Bauman predicted bonds to others to become progressively ‘loosely tied’ and ‘easy to untie’, [28] Beck and Beck-Gernsheim emphasized how individuals seek increasing autonomy as a risk-minimization strategy, [29] while Giddens noted that partnerships are progressively incorporating equality and democracy as core values. [30] As Lauer and Yodanis put it: “*within individualized marriage*, *the two individuals are less likely to become one interdependent unit or to sacrifice their own individuality for socially defined roles*. *They pursue their own interests and goals”*. [12] Similar tensions within families, marriages and cohabiting relationships between individualism on the one hand, and commitment to the group on the other, have been noted by family scholars [7,10,11,31]. Yodanis and Lauer eloquently articulate the conceptual relationships between domestic money and the individualization thesis: *“If marriage were individualized*, *we would expect married couples to be more likely […] to act in ways that maintained their independence and individual identity […] spouses should be more likely to do things like […] keep their resources separate*, *including having separate bank accounts”*. [32] While this quote refers explicitly to marriages, the same reasoning may apply also to long-term cohabiting unions. In reviewing the existing literature, Yodanis and Lauer noted that couples still tend to pool their resources into a “common pot”, are only slightly more likely over time to keep and manage individually earned money separately, and switch from having separate finances to pooling their resources shortly after marriage. [32] These empirical regularities were collectively taken as evidence against the marriage individualization thesis, but the authors noted incipient trends towards individualization in the financial domain that require further attention. Examining continuity and change over time in the banking arrangements of Australian couples is one of the aims of the present study.

### 2.2 The “common pot” vs “separate purses”: The symbolic and practical importance of the joint account

Understanding how couples manage money is critical in assessing tensions between individual autonomy and collective interests [2,3,7,13,33]. Collectivist approaches attribute value to the communal sharing of resources as a way of enhancing group functioning, solidarity and purpose [13]. Joint bank accounts are a collectivist “common pot” approach to financial management, whereby couples merge their individual interests into a single economic collective [7]. They minimize exchange costs between family members, contribute to strengthening family bonds, and signal commitment by individual partners to the conjugal family [3,7,13]. At the other end of the spectrum are utilitarian or privatized “separate purses” approaches to financial management based on individualistic principles, whereby partners preserve distinct property rights by ‘holding resources back’ [7]. In this approach, best exemplified by the presence of separate bank accounts, material autonomy and self-interest prime over collective goals, and goods and services are exchanged between partners following market-like relationships [1,3,7,13,16].

It follows that the joint account carries both symbolic meaning and practical implications for individuals and couples. Concerning its symbolic meaning, the joint account is often regarded as the figurative expression of “trust” and “mutual commitment” in marriages and cohabiting unions, reflected by the absence of formal accounting and monitoring of domestic (or marriage) money [2,6,34]. Joint accounts are also instrumental for partnerships to reconcile frictions between the egalitarian ideology that money in marriage should be shared equally regardless of who brings it into the household, the individualistic ideology that people own the money they earn, and the reality that partnered men are still much more likely than partnered women to bring (larger amounts of) money into the household [4,24,35]. This is accomplished by stressing ‘jointness’ (signifying ‘equity’) rather than ‘equality’ as a guiding principle in intra-household financial management [2,5]. The joint account acts as an equalizing factor within relationships: it denies shares, makes the individual collective, renders individual economic contributions to the household less visible, and makes individually earned money become joint money [2,36]. By the same token, the joint account helps block questions and discussions about equality, power and control [2,5].

Concerning its practical implications, the joint account offers both couple members comparable information on and access to money, both of which are necessary preconditions for exerting control over money [2,3,5,6]. This contrasts with situations in which only one partner (usually the man) manages all assets, which are documented to lead to imbalances in money control, personal spending and individual experiences of deprivation within partnership [3]. It has also been suggested, and occasionally empirically tested, that intra-household organization models may have consequences for many aspects of individual and family life. In this regard, joint management models are generally thought of as leading onto better and more democratic outcomes than separate management models on domains such as relationship adjustment [37] and quality [13], financial satisfaction [15], mental health [14], family relationships [38], marital happiness [9], and the ability of partners to leave unhealthy or unsafe unions [39]. On the other hand, some have argued that financial independence via separate banking may be an important factor safeguarding women’s economic futures in the event of union dissolution [14].

In sum, emerging from this literature is the notion that holding joint vs. separate accounts is a factor of symbolic and practical importance to individuals and couples. Thus, identifying the characteristics of those couples following collectivistic vs. individualistic financial organization models is a worthwhile endeavor. In our analyses, we grapple with these issues empirically.

### 2.3 Gender, power and within-household financial management

Significant efforts have been devoted to theorizing the complex interplay between money, power and gender within households, and how these different factors intersect with intra-household financial arrangements–including banking arrangements [4,8,9]. Key concepts include control (the ability to influence decisions), management (the implementation of decisions already made), and power (resulting from the combination of control and management) [40]. It is however recognized that these are partially overlapping concepts with blurry boundaries [4], that their connections and causal ordering are unclear [9], and that other concepts–such as access, knowledge or consumption–are also relevant [15,41,42]. A key distinction is that between *strategic control* over how resources are spent (operationalized via decision-making variables in empirical studies) and the day-to-day executive management of resources (operationalized as banking arrangements) [15,23,33]. This implies that banking arrangements cannot be taken as a direct *proxy* for financial control or power within the household [2], although it is clear that they are associated with different degrees of gender equality in financial control [4,23].

The symbolic and practical implications of “common pot” and “separate purses” approaches to the management of domestic money need to be understood in a broader, highly gendered context. The study of money is in fact central to feminist concerns, including power, equality, independence and choice [2]. As women’s economic contributions to the household have been historically smaller than men’s, families had to devise systems of resource allocation and management [4]. The *resource theory of power* was one of the first attempts to explain gender inequalities in financial arrangements within the household, proposing that the spouse bringing the most resources to a partnership should hold the most power over how such resources are managed and spent [9,43]. While this theory originated in the 1960s, the reality it depicts still applies to many households in developed countries. For example, in 70% of heterosexual households in the Australian data used in our analyses, the male partner out-earns the female partner. The resource theory of power has nevertheless been criticized for placing the onus on how money *enters* the household, but failing to account for how money is *handled* within the household [4,9].

The *intra-household economy perspective* devised by Pahl and Vogler in the 1980s focused on the latter, classifying couples depending on the system they used to organize their money [21,22]. In the *whole wage* system, the man hands his full earnings to the woman to manage; in the *housekeeping allowance* system, the man gives the woman an allowance to confront family expenditure and keeps the rest of the money; in the *pooled management* system, both partners put money into a shared account and use the money as needed; and in the *independent management* system, partners have both separate control over income and responsibility for expenditures [4,16]. Modified or refined versions of this typology have been formally proposed [27] and used in practice [7]. A factor stressed by Pahl and Vogler’s approach is that focusing on how money is handled, rather than how it enters the household, is important. As Yodanis and Lauer [3] explain, couples’ decisions to pool their income in a common pot versus maintaining separate purses is an important indicator of the level of investment and integration in their intimate relationship, as well as the level of equality in such relationship. Because ideological and cultural values affect the choice of management system, gender inequalities in economic well-being may be more strongly tied to women’s access to money than their relative income [15]. Consistent with this, different intra-household economy models have been shown to exacerbate or diminish within-household gender inequalities in decision-making power and living standards [9,24]. Generally, joint pooling is associated with the most gender egalitarian outcomes concerning money control and living standards [9,15], whereas women are disadvantaged if one spouse, either the wife or the husband, manages assets [3,26,44].

## 3 Predictors of couples’ banking arrangements

The empirical analyses within this study are devoted to improving our understanding of couples’ banking arrangements, a task which only a handful of studies using robust quantitative methods have previously undertaken. These include Treas [7] and Kenney [17] for the US, Cheal [45] for Canada, Kan and Laurie [14] for the UK, Lyngstad, Noack and Tufte [1] for Norway, and Lee and Pocock [46] for South Korea. In these studies, banking arrangements were found to be associated with couple members’ absolute income, education, ethnicity, employment status, marital status, relationship quality and family size. Taking together these previous findings, and theory and evidence from the broader literature on within-couple financial organization and practices, we derive testable hypotheses about how other factors not previously considered in the literature (or only partially considered) should relate to couples’ banking arrangements in our contemporary Australian panel data.

### 3.1 Absolute and relative income

Income has been found to be a key predictor of within-couple financial arrangements [3,16,22]. The effect of income on financial arrangements depends on two factors. The first factor is the position of couples’ total income in the income distribution. Independent financial arrangements are more prevalent among high-income couples, whereas resource sharing occurs more often in low- and moderate-income couples [27]. This may be because resource pooling accomplishes economies of scale in household production, whereas high-income couples are able to forgo the cost advantages by adopting multiple bank accounts (i.e., separate accounts) in pursuit of financial autonomy. The second factor is the relative contributions of the male and female partners to couples’ total income (i.e., their relative resources). Relative resources and bargaining power theories pose that an individual’s power in household decision making is proportional to the amount of resources that she/he contributes to the household *vis-à-vis* her/his partner [47]. Particularly, couples are more likely to pool resources as the male and female income contributions approach equality, and more likely to bank separately when women contribute more than men to household income [3,17]. This resonates with research findings indicating that financial independence is higher in dual-earner couples [25,27]. Based on this literature, we hypothesize that:

H1a: Couples’ total income will be positively associated with the probability of holding separate bank accounts.

H1b: Unequal contributions to household income will be associated with separate banking strategies, with women’s contributions being more predictive of separate bank accounts than men’s contributions.

### 3.2 Children as a transaction cost

The resources necessary to raise children (e.g., time and effort) are scarce, and so families with children operate subject to constraints. In this context, families must strategically allocate their finite resources to maximize outputs. One way to accomplish this is smoothing their daily operations by minimizing everyday-life hassles, constant auditing of the spending of the other couple member and persistent negotiations on what money needs to be spent on. In the context of family finance research, the presence of children has been argued to increase ‘transaction costs’, i.e., costs associated with bargaining and monitoring household resource spending among couple members [7]. For example, children increase the number of payments and daily financial operations within households. To minimize these transaction costs, couples with children will be particularly likely to seek efficient banking strategies that enable them to maximize personal and household utilities. Specifically, having joint bank accounts should reduce time-consuming discussions and negotiations about whose account to use to make payments. We therefore hypothesize that:

H2: The number of dependent children in the household will be positively associated with the likelihood of having a joint bank account.

### 3.3 Relationship history and duration

We argue that relationship history should predict financial organization because, compared to other couples, remarried/re-partnered couples are more likely to consider money management as a major issue in their relationship, may have gained a certain degree of financial autonomy, and may have more complicated financial situations, e.g., they may retain complex financial links with their ex-partners and/or biological children [5,13,16,48]. Collectively, these suggest that remarried/re-partnered couples may have a tendency towards banking separately. Conversely, couples in longer relationships may be more likely to bank jointly, because the longevity in their relationship is indicative of mutual trust. Empirically, evidence suggests that relationship duration is positively associated with the likelihood of income pooling [1]. On the other hand, the incidence of separate financial management in remarried couples is much higher than in the general population. This suggests that resource pooling in new families is hampered by unresolved financial problems from previous relationships, the desire to protect one’s financial assets in case the new relationship breaks down, or as an ‘exit’ option from such relationship [48]. Studies also indicate that having a previous history of union dissolution predicts banking arrangements: couples in which at least one partner was divorced or widowed are less likely to use joint bank accounts [7,16]. Based on the existing theory and evidence, we hypothesize that:

H3a: Remarried/re-partnered couples will be more likely to use separate bank accounts than couples in their first marriages/de facto relationships.

H3b: Relationship duration will be positively associated with the probability of resource pooling.

### 3.4 Gender-role attitudes

Since gender attitudes are often predictive of subsequent behavior [49], these can be an important driver of within-couple financial arrangements [50]. The perception that women should prioritize homemaking and childrearing justifies men’s assertion of masculinity and domination in household money control and financial decision making, which should in turn preclude financial separateness—particularly for women. Consistent with this, traditional gender ideology is associated with joint access to money and authoritarian control over money by the male partner [17]. In contrast, the egalitarian ideology of co-providing emphasizes independent money control and management [9,27,45]. We therefore hypothesize that:

H4: Couples in which partners hold traditional gender-role attitudes will be less likely to have separate bank accounts than couples in which partners hold egalitarian gender-role attitudes.

### 3.5 Intergenerational effects

The family is a socializing unit through which children learn about their social world. One component of this socialization process is the transmission of information, attitudes, values, etc. about money and finances from parents to children. Through explicit education, information sharing, and day-to-day interactions, parents pass onto their children financial attitudes, knowledge and capabilities [51,52]. This financial mentality is then brought into intimate relationships, and enacted—among others—via the choice of banking arrangements. Family financial socialization theory suggests that individuals’ financial perceptions and practices are reflective of parental social class and parental education [51,52]. Specifically, high parental education and occupational status are associated with prudent saving, rational spending and strategic financial planning [51,53]. Additionally, highly educated parents are more likely to set up egalitarian family arrangements concerning finances and money management [54]. Hence, it is plausible that their adult children also do so through the impact of socialization and role modelling. We thus predict that:

H5a: Couples in which partners come from high socio-economic family backgrounds will be more likely to organize money separately than couples in which partners come from low socio-economic family backgrounds.

Another strand of intergenerational research on the transmission of financial attitudes and practices has focused on the role of gender egalitarian attitudes and practices in the parental generation. For example, growing up in a family in which the mother held egalitarian gender-role attitudes has a large positive effect on daughters’ gender ideology and labor market outcomes, such as the probability of full-time employment and work hours [55]. Other research has found similar results for maternal engagement in the labor force, earnings and occupational standing [56,57]. These families can be described as ‘female-empowered families’, in the sense that mothers’ bargaining power is more comparable to fathers’. Children raised in such families (particularly daughters) are likely to emulate these arrangements as adults, which should in turn translate into egalitarian financial arrangements by banking separately as they form their own family. While research on this is limited, descriptive analyses reveal substantial intergenerational continuity in money management among couples in the UK, whereby adult children’s financial management resembles that of the parental generation [22]. Based on this, we expect that:

H5b: Couples in which partners come from female-empowered family backgrounds will be more likely to organize money separately than couples in which partners come from other family backgrounds.

In the next section we introduce the data and methods used in our empirical analyses.

## 4 Data and methods

### 4.1 Dataset

We use data from the Household, Income and Labour Dynamics in Australia (HILDA) Survey, a nationally representative panel survey initiated in 2001 with 13,969 respondents from 7,682 households. This includes a top-up sample consisting of roughly 4,000 individuals added to the survey in 2011. Data were collected primarily via face-to-face interviews and self-complete questionnaires with in-scope respondents aged 15 years and over residing in private dwellings. Since then, interviews have been conducted annually. New individuals can join the panel if they live in participating households and turn 15 years of age, or if they begin a relationship or have a child with an original sample member. The HILDA Survey has relatively high wave-on-wave response rates ranging from 86.8% in wave two to 96.5% in wave 14 [58]. This survey is unique for our research purposes for several reasons. First, its wealth module collects longitudinal information on participants’ bank account ownership in four occasions: wave 2 (2002), wave 6 (2006), wave 10 (2010) and wave 14 (2014). Few international panel surveys collect such complex information over an extended period of time and on an ongoing basis. Second, data on joint bank accounts contain personal identifiers of household members, which enables us to determine whether a joint bank account is held by both couple members. Third, couple-level data enable more precise estimates of the effects of relative income and relationship history than individual-level data. The power and usefulness of the HILDA Survey to answer research questions pertaining to couple relationships and financial arrangements is exemplified by previous studies—for instance, recent analyses by Fulda and Lersch examining whether and how individuals change financial planning horizons as they change partnership status [59].

### 4.2 Information on banking arrangements

In the HILDA Survey’s wealth module, respondents are asked whether they have any bank accounts in their name only (i.e., separate accounts), and whether they hold any joint bank accounts with other people (i.e., joint accounts). For respondents who indicated that they hold joint bank accounts, the number of joint accounts and the identity of other household members who co-held each of the accounts were asked. We use this information to identify different banking arrangements at the couple level, and create two outcome variables. First, we construct a dichotomous variable indicating whether or not couples have at least one joint account. Second, we construct a five-category variable splitting couples as follows: (i) both partners have a joint bank account only (i.e., no separate bank accounts); (ii) both partners have a joint bank account & only the male partner has a separate bank account; (iii) both partners have a joint bank account & only the female partner has a separate bank account; (iv) both the male and female partners have a joint account & separate bank accounts; and (v) both partners have separate bank accounts, but no joint account. For 4.27% of couples (*n* = 1,336) there are mismatches in partners’ reports of joint bank accounts. Most of these emerge when one partner reports having a joint bank account, but the other partner does not. In these cases, we consider couples as having a joint bank account as long as one partner indicates so.

### 4.3 Sample selection

Our initial sample includes 34,785 observations from 15,578 *partnered* individuals with valid bank account information. For theoretical reasons, we drop person-year observations from (i) individuals who did not cohabit with their partners (*n* = 88), (ii) from second and higher-order partnerships if respondents did not have a consistent partner over the observation period (*n* = 638), (iii) same-sex couples (*n* = 312), and (iv) couples in which either partner reported not having a bank account (*n* = 480). For reasons related to data quality, we drop person-year observations from (i) individuals whose partners did not participate in the survey (*n* = 1,827), (ii) couples in which the male and female partners reported inconsistent marital statuses (*n* = 18), and (iii) couples in which either partner had missing data on variables used as model controls (*n* = 26). Missing data is comparatively larger for some of the key explanatory variables due to survey design–particularly gender-role attitudes (*n* = 1,298 observations) and relationship duration (*n* = 116 observations). Therefore, we choose to only exclude observations with missing data on such variables from the models in which these variables are used. Based on data from the matched couples, we derive couple-level analytical variables by using information from both couple members, resulting in two identical records for each couple (*n* = 30,758). We retain only one of these two identical records. Our final analytical sample consists of 15,379 observations from 7,054 couples. This is an unbalanced panel with an average of 2.9 occurrences per couple over the 4 observation points.

### 4.4 Key explanatory variables

The key explanatory variables used in our analyses represent factors hypothesized to affect couples’ banking arrangements, and are derived as follows.

#### Income

For total income, we take the inverse hyperbolic sine (IHS) transformation of the sum of both partners’ financial-year gross total incomes, after having adjusted these for inflation to 2014 prices using annual Consumer Price Index rates. We apply the IHS transformation to the income measure because this transformation can effectively deal with non-positive income values while, at the same time, transforming a positively skewed income distribution into a normal distribution. The formula for the IHS transformation is $\text{log}\left(income+\sqrt{income^{2}+1}\right)$. For relative income, we create a categorical variable with three categories: (i) women contribute 60–100% of the income (i.e., men contribute 0–40% of the income); (ii) both men and women contribute 40–60% of the income; and (iii) men contribute 60–100% of the income (i.e., women contribute 0–40% of the income). We choose these specific thresholds to make our results comparable to those of previous studies using the same thresholds [17]. This approach is also a means of contrasting a situation of ‘broad equality’ (40–60% of the household income earned by both partners) to different types of ‘gender inequality’ (either the man or the woman earns a majority of the household income). Yet this choice is somewhat arbitrary. To test the robustness of the results, in sensitivity analyses (not reported) we also considered specifications in which the thresholds used to determine spousal income contributions were defined as 35%/65%—instead of 40%/60%. The pattern of results, available from the authors upon request, is highly consistent.

#### Number of children

In the HILDA Survey, dependent children are defined as persons under 15 years of age, or persons aged 15–24, who are engaged in full-time study, not employed full-time, living with one or both parents, not living with a partner, and who do not have a resident child of their own. There are four existing variables in the HILDA Survey dataset, identifying the number of dependent children aged 0–4 years, 5–9 years, 10–14 years and 15–24 years. We aggregate these four available variables to create a single continuous variable measuring the number of dependent children in the household.

#### Relationship history and duration

The HILDA Survey contains information on the number of marriages and *de facto* relationships (of 3 months or more) participants have ever had, from a life cycle perspective. This variable uses information from a HILDA Survey question asking new respondents:*“How many times*, *in total*, *have you been legally married*? *That is*, *in a registered marriage*. *Include your [current / last] marriage”* to calculate the number of previous marriages. It also uses information from HILDA Survey questions asking new respondents:*“Some people live together as a couple without marrying*. *Have you ever lived with someone as a couple for more than three months*, *but did not marry them*?*”* and *“How many such relationships have you lived in*?*”* to calculate the number of previous *de facto* relationships. This information is then automatically updated by the survey managers to incorporate any additional marriages or *de facto* relationships observed while individuals remain part of the study. We use this in combination with respondents’ current marital status to separate individuals who are in their first marriage/*de facto* relationship from individuals who are in their second or higher order marriage/*de facto* relationship. At the couple level, we combine this information from both partners into a variable containing four categories: (i) both partners are in their first marriage/*de facto* relationship; (ii) men are in their first marriage/*de facto* relationship and women in their second or higher order marriage/*de facto* relationship; (iii) women are in their first marriage/*de facto* relationship and men in their second or higher order marriage/*de facto* relationship; and (iv) both partners are in their second or higher order marriage/*de facto* relationship. Relationship duration for both marriages and *de facto* relationships is recorded in years.

#### Gender-role attitudes

In its self-complete questionnaires, the HILDA Survey asks about respondents’ gender-role attitudes. These questions were included in waves 1, 5, 8 and 11, with responses being carried forward to the closest posterior wave with information on bank accounts. We use the degree of respondents’ agreement (on a scale from 1 to 7) with the following four items to measure respondents’ attitudes towards gender roles: (i) *“Many working mothers seem to care more about being successful at work than meeting the needs of their children*”; (ii) *“Whatever career a woman may have*, *her most important role in life is still that of being a mother”*; (iii) *“Mothers who don’t really need the money shouldn’t work”*; and (iv) *“It is better for everyone involved if the man earns the money and the woman takes care of the home and children”*. We choose these four items out of a wider pool because they maximize measurement reliability (Cronbach α = 0.7). Higher scores represent more traditional attitudes, with variables being reverse coded where necessary. Scores in each of these items are then summed and rescaled to create an index ranging from 0 (most egalitarian attitudes) to 100 (most traditional attitudes). This requires the following transformation: new score = (old score − 4) / (28 − 4) * 100. We then create a variable measuring the average attitude index score of each couple, by taking the mean of both partners’ scores.

#### Family background

Parental occupation and education are used to capture the socio-economic status of the family in which respondents grew up. Parental occupational status is measured by the Australian Socioeconomic Index 2006 [60], while parental education is recoded into three categories: (i) school year 12 and below, (ii) professional qualification, and (iii) bachelor degree or higher. We create a continuous variable measuring the average status of the family by taking the mean occupational status scores of parents. In addition, we derive a dichotomous variable identifying whether the respondent comes from a ‘female-empowered family’, i.e., a family in which the mother’s educational level is higher than or equal to the father’s educational level. We then create a couple-level categorical variable comparing the partners’ family background: (i) both partners come from female-empowered families; (ii) only the male partner comes from female-empowered families; (iii) only the female partner comes from female-empowered families; and (iv) neither partner comes from a female-empowered family.

#### Control variables

Our models control for other known predictors of couples’ banking arrangements, based on previous studies [1,7,14,17,45,46]. Given that married and cohabiting couples tend to display different financial arrangements [6,33] and financial planning horizons [59], we separate married couples from couples in *de facto* relationships through a ‘married’ dummy variable. In the HILDA Survey, marital status is derived from responses to questions on registered marital status and (if not married) whether living with someone in a relationship. In this study, individuals are considered to be married if they are in a registered marriage at the time of the interview. Individuals in a *de facto* relationship are those who, at the time of interview, are never married, separated, divorced or widowed and are in a relationship with someone to whom they are not married. Mean age is a continuous variable capturing the average age of both couple members (expressed in years), while the age gap is captured by a trichotomous categorical variable: (i) men are at least 5 years older than women; (ii) the age difference is within five years; and (iii) women are at least 5 years older than men. Controls for couple-level education are measured through a variable capturing the following scenarios: (i) both partners have University degrees; (ii) only the male partner has a University degree; (iii) only the female partner has a University degree; and (iv) neither partner has a University degree. We also control for couples’ employment status: (i) both partners are employed; (ii) only the male partner is employed; (iii) only the female partner is employed; and (iv) neither partner is employed. Because there are cultural differences in financial practices [5], we also control for ethnicity in our models. We use a variable that separates couples into those in which (i) both partners were born in Australia; (ii) only the male partner was born in Australia; (iii) only the female partner was born in Australia; and (iv) neither partner was born in Australia.

Descriptive statistics for the complete sample and stratified by bank account choices are presented in Table 1.

**Table 1:** Sample descriptive statistics.

	Number of observations	All couples	Banking arrangements				
			Joint only	Joint + man separate	Joint + woman separate	Joint + both separate	Both separate only
*Dependent variable*	15,379	n/a	31.3	7.7	16.8	23.3	20.9
*Key independent variables*							
Total income, HIS transformation ^a^	15,379	12.1 (0.9)	12.0 (0.9)	12.2 (0.7)	12.1 (0.9)	12.2 (0.8)	11.9 (0.8)
Relative income, % ^b^	15,379						
Women contribute 60%+		12.5	26.0	7.0	18.0	24.6	24.5
Similar income contributions		36.7	34.5	6.8	14.4	23.1	21.2
Men contribute 60%+		50.8	30.3	8.5	18.3	23.2	19.8
Dependent children, number ^a^	15,379	0.9 (1.2)	0.9 (1.2)	1.1 (1.2)	1.0 (1.2)	0.9 (1.1)	0.8 (1.1)
Relationship duration, years ^a^	15,263	19.8 (16.4)	25.3 (16.4)	19.9 (15.1)	23.5 (15.3)	16.8 (15.5)	12.0 (14.9)
Relationship history, % ^b^	15,378						
Both 1^st^ relationship		64.5	38.6	8.8	19.2	21.4	12.0
Men 1^st^ relationship and women 2^nd^+		5.5	27.8	6.3	20.2	22.9	22.9
Women 1^st^ relationship and men 2^nd^+		6.1	29.1	9.2	16.1	24.8	20.8
Both 2^nd^+ relationship		23.9	12.8	4.6	9.8	28.2	44.6
Gender-role attitudes, mean score (0–100) ^a^	14,081	54.7 (14.1)	56.9 (14.0)	55.3 (14.0)	55.5 (13.6)	52.3 (13.6)	53.3 (14.7)
Parental socio-economic status, mean (0–100) ^a^	15,366	42.3 (15.8)	40.8 (15.0)	44.0 (16.3)	41.0 (15.1)	44.7 (16.2)	42.3 (16.3)
Female-empowered family background, %	15,379						
Both female-empowered family		35.1	31.7	7.3	16.8	22.8	21.4
Only man female-empowered family		15.0	30.1	8.2	16.5	25.2	20.0
Only woman female-empowered family		16.7	30.1	8.8	17.8	25.2	18.1
Neither female-empowered family		8.8	33.5	8.0	16.1	25.2	17.2
*Controls*							
Marital status, % ^b^	15,379						
Married		81.4	7.5	4.3	6.2	29.0	53.1
*De facto* relationship		18.6	36.7	8.5	19.2	22.0	13.5
Mean age, years ^a^	15,379	47.5 (15.6)	51.3 (15.3)	46.7 (14.2)	50.3 (14.0)	45.7 (15.2)	42.0 (16.5)
Age difference, % ^b^	15,379						
Man 5+ years older		18.9	28.8	7.7	14.3	23.6	25.5
Age difference within 5 years		78.1	32.2	7.8	17.5	23.1	19.4
Woman 5+ years older		3.0	22.3	5.2	14.0	27.0	31.5
Employment status, % ^b^	15,379						
Both employed		55.0	28.9	7.9	17.0	26.7	19.5
Only man employed		18.9	29.7	9.7	17.4	20.7	22.5
Only woman employed		4.9	24.3	6.8	16.5	23.5	28.9
Neither employed		21.3	40.4	5.7	15.9	17.0	21.2
Education, % ^b^	15,379						
Both have degree		14.6	26.6	10.3	15.7	29.8	17.6
Only man has degree		10.1	30.8	8.6	16.8	27.0	16.8
Only woman has degree		12.1	30.4	8.9	14.5	26.0	20.3
Neither has degree		63.2	32.6	6.7	17.5	20.8	22.4
Ethnicity, % ^b^	15,379						
Both born in Australia		65.9	30.2	7.2	17.4	23.5	21.7
Men born in Australia only		8.9	26.1	9.0	16.0	28.9	20.1
Women born in Australia only		10.5	30.7	8.1	16.1	25.4	19.7
Neither born in Australia		14.7	39.6	8.7	15.2	17.8	18.7

HILDA Survey (2002, 2006, 2010 & 2014). Sample mean for continuous variables and percentages for categorical variables reported. Standard deviations in parentheses.

^a^ *p*<0.05 in a one-way ANOVA test.

^b^ *p*<0.05 in a Chi-square test.

### 4.5 Statistical modelling

We extend Treas’s analyses of couples’ banking arrangements [7] using panel data and panel regression models. Unlike cross-sectional techniques, panel models take into consideration both within-couple and between-couple differences in banking arrangements over time, improving efficiency and reducing bias in their predictions of the longitudinal associations between our factors of interest and couples’ banking arrangements. A key advantage of using panel data compared to cross-sectional data is the ability to leverage multi-year data to incorporate in the statistical estimation changes in couple’s banking behavior over time. This is important, because cross-sectional data can only capture group differences between couples at one point in time, and so are limited in understanding how couples compare themselves across different time points. Panel data enable comparisons within and between couples, thereby providing more accurate and efficient statistical estimates than cross-sectional data.

First, we estimate a set of couple-level random-effect binary logit models that predict whether or not couples hold a joint bank account. These models are extensions of cross-sectional binary logit models for panel data. Let $\eta_{ct}$ denote the ratio of the probability of having a joint bank account ($\pi_{ct}$) to the probability of not having a joint bank account for couple c at time $t,\;X_{ct}$ denote time-varying variables, $Z_{c}$ denotes time-invariant variables, and $u_{c}$ refers to a couple-level random effect. This gives the following random-effect logit model for panel data:

$$\log\left(\eta_{ct}\right)=\log\left(\frac{\pi_{ct}}{1-\pi_{ct}}\right)=\beta^{\prime }X_{ct}+\theta^{\prime }Z_{c}+u_{c}$$ (1)

Second, we estimate a set of couple-level random-effect multinomial logit models that distinguish between five different types of banking arrangements. Let ${\tilde{\pi }}_{ct}^{\left(j\right)}$ and ${\tilde{\pi }}_{ct}^{\left(b\right)}$ denote the probability of the banking arrangement falling into the j^th^ category and the reference category respectively for couple c at time $t,\;\beta_{j}$ denote the jth coefficient vector associated with $X_{ct}$, and $\theta_{j}$ denote the jth coefficient vector associated with $Z_{c}$. This gives the following random-effect multinomial logit model for panel data:

$$\text{log}\left(\frac{{\tilde{\pi }}_{ct}^{\left(j\right)}}{{\tilde{\pi }}_{ct}^{\left(b\right)}}\right)=\beta_{j}^{\text{'}}X_{ct}+\theta_{j}^{\text{'}}Z_{c}+u_{c},\;j\neq b$$ (2)

We express the results of random-effect logit and multinomial logit models as odds ratios.

## 5 Empirical evidence

### 5.1 Prevalence of and trends in banking arrangements in contemporary Australia

**Fig 1:**
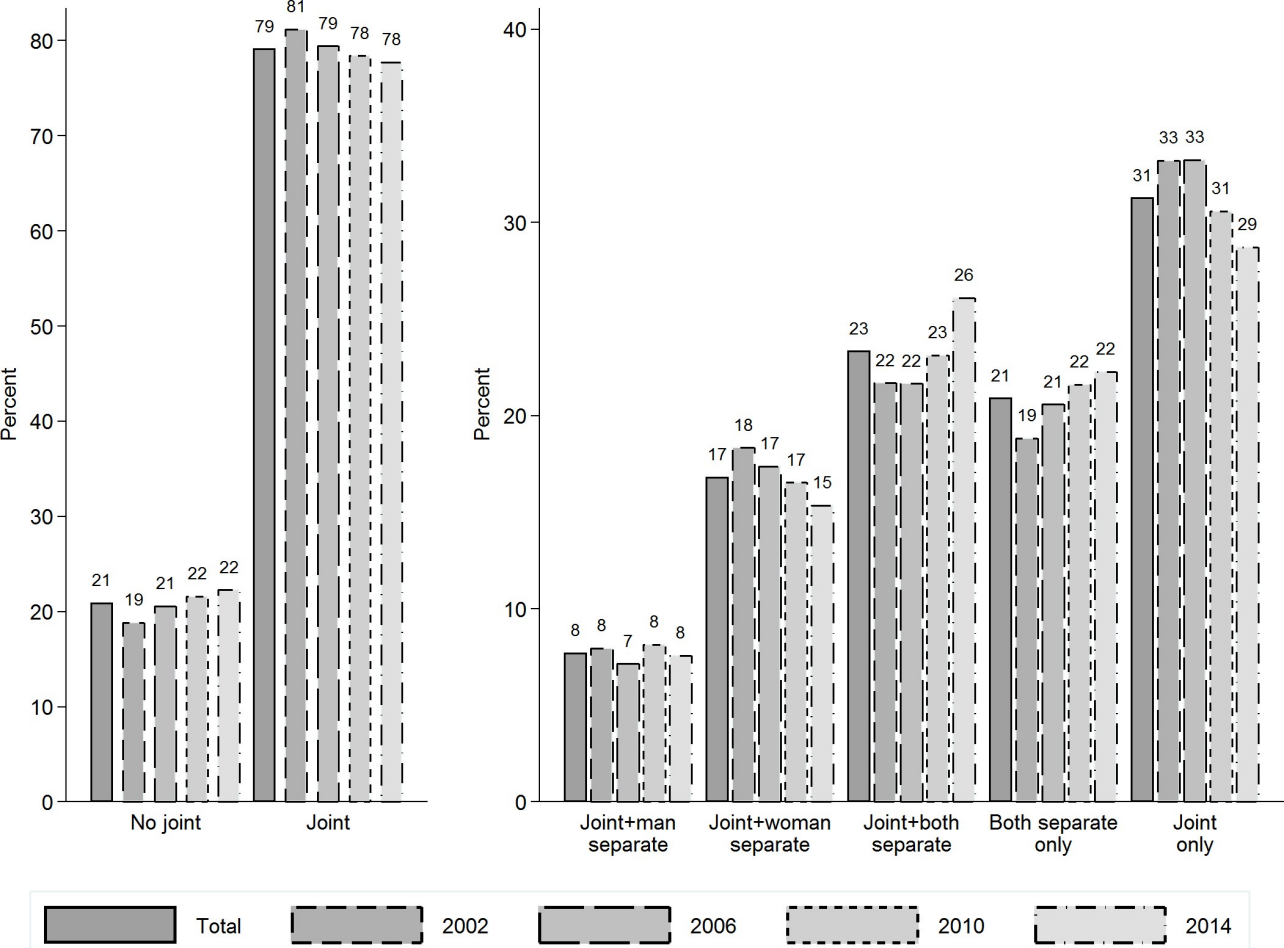
Trends over time in banking arrangements of heterosexual couples in Australia.

Fig 1 shows the distributions of our two outcome variables capturing couples’ bank account arrangements, across all survey years and over time. Results for the dichotomous outcome variable indicate that *circa* 79% of couples in Australia have joint bank accounts and the remaining 21% do not have joint accounts. These percentages remained rather stable over the observation window. Nevertheless, formal tests reveal that the difference between the first wave (Wave 2, 2002) and the last wave (Wave 14, 2014)—a change of 3 percentage points—is statistically significant (*p*<0.001).

HILDA Survey (2002, 2006, 2010 & 2014). Pooled observations from all couples. n(observations): 15,379; n(couples): 7,054.

Results for the five-category outcome variable indicate that, overall, 31% of couples have joint accounts only (i.e., no separate accounts), 23% have joint accounts plus separate accounts for both partners, 21% have separate accounts for both partners but no joint account, 17% have joint accounts plus a separate account for the female partner, and 8% have joint accounts plus a separate account for the male partner. The gender difference in the last two categories is somewhat surprising, as will be discussed later. There is also more over-time variation in the distribution of this five-category variable. Between 2002 and 2014 there were increases in the percentage of couples in which both partners have joint and separate bank accounts (from 22% to 26%) and only separate bank accounts (from 19% to 22%), and a decrease in the percentage of couples relying exclusively on a joint account (from 33% to 29%). Formal tests indicate that all of these differences are statistically significant (*p*<0.001). This suggests that, as time unfolds, there is increasing financial separateness among individuals within heterosexual couples in Australia.

### 5.2 Factors predicting couples’ banking arrangements

Table 2 presents the results of a series of couple-level panel regression models aimed at testing our research hypotheses.

**Table 2:** Banking arrangements among heterosexual couples in Australia, odds ratios.

	Joint account vs. no joint account	Banking arrangements (ref. partners have only a joint account)			
		Joint+man separate	Joint+woman separate	Joint+both separate	Both separate only
*Hypothesis 1* ^a^					
Total income (IHS)	1.31^***^	1.29^***^	1.08	1.30^***^	0.94
Relative resources (ref. similar contribution)					
Women contribute 60%+	0.74^*^	1.30	1.59^***^	1.39^**^	1.59^***^
Men contribute 60%+	1.06	1.11	1.29^**^	1.00	1.07
N (observations)	15,379	15,379			
N (couples)	7,054	7,054			
AIC/BIC	11,158/11,295	40,944/41,471			
*Hypothesis 2* ^b^					
Number of dependent children	1.32^***^	0.88^**^	0.92	0.77^***^	0.76^***^
N (observations)	15,379	15,379			
N (couples)	7,054	7,054			
AIC/BIC	11,130/11,260	40,872/41,369			
*Hypothesis 3* ^c^					
Relationship history (ref. both 1^st^ relationship)					
Men 1^st^ relationship and women 2^nd^+	0.22^***^	1.44	2.02^***^	1.67^*^	2.81^***^
Women 1^st^ relationship and men 2^nd^+	0.32^***^	1.82^*^	1.63^*^	1.75^**^	2.32^***^
Both 2^nd^+ relationship	0.04^***^	5.16^***^	5.34^***^	9.81^***^	21.73^***^
Relationship duration	1.07^***^	0.98^*^	0.97^***^	0.95^***^	0.94^***^
N (observations)	15,263	15,263			
N (couples)	7,006	7,006			
AIC/BIC	11,084/11,229	40,707/41,264			
*Hypothesis 4* ^b^					
Gender-role attitudes	1.00	1.00	0.99	0.99^***^	0.99^*^
N (observations)	14,081	14,081			
N (couples)	6,489	6,489			
AIC/BIC	10,014/10,142	37,726/38,217			
*Hypothesis 5* ^b^					
Mean parental socio-economic status	1.00	1.01	1.00	1.01^*^	1.00
Family background (ref. neither from female-empowered family)					
Only man from female-empowered family	0.66	1.31	1.28	1.28	1.44
Only woman from female-empowered family	0.81	1.48	1.49	1.39	1.48
Both from female-empowered family	0.56^*^	1.24	1.33	1.26	1.67^*^
N (observations)	15,366	15,366			
N (couples)	7,044	7,044			
AIC/BIC	11,145/11,305	40,926/41,544			

HILDA Survey (2002, 2006, 2010 & 2014). Column 1: random-effect binary logit models. Columns 2–4: random-effect multinomial logit models. All models feature robust standard errors.

^a^ controls: marital status, age, employment, education and ethnicity.

^b^ controls: marital status, age, employment, education, ethnicity and total income (IHS).

^c^ controls: age, employment, education, ethnicity and total income.

^*^ *p<*0.05,

^**^ *p<*0.01,

^***^ *p<*0.001. Complete tables of model coefficients are available from the authors upon request.

#### 5.2.1 Absolute and relative income

The first set of models in Table 2 examines the associations between absolute and relative income and couples’ banking arrangements. In the random-effect logit model absolute income is associated with an increase in the odds of couples choosing a joint account (OR = 1.31, *p<*0.001). Nevertheless, results from the random-effect multinomial logit model reveal that it actually raises the odds that couples have some combination involving separate accounts. These seemingly contradictory results resonate with findings from Treas [7]: while partially pooling their resources, high-income couples also maintain a certain extent of financial autonomy to ensure freedom in personal spending. This is not evident in the simpler binary logit model, as joint accounts are often accompanied by separate accounts. Consistent with our relative resources hypothesis, we find that couples in which the female partner contributes more income to the household have significantly lower odds of having a joint account than couples in which both partners make similar income contributions (OR = 0.74, *p<*0.05). The random-effect multinomial logit model results further reveal that greater income contributions to the household by the female partner are associated with increased odds that couples have any bank account arrangement involving separate accounts, suggesting that women’s income contribution to the household is more predictive of separate banking than men’s.

#### 5.2.2 Children as a transaction cost

The second set of models in Table 2 examines the associations between the number of dependent children in the household and couples’ banking arrangements. In the random-effect logit model, additional children are associated with increased odds of having a joint account (OR = 1.32, *p<*0.001). In the random-effect multinomial logit model, children are associated with reduced odds of having separate accounts for men or both partners (OR_men_sep_ = 0.88, *p<*0.01; OR_both_sep+joint_ = 0.77, OR_sep_only_ = 0.76, *p<*0.001). These findings are consistent with our second hypothesis, and suggest that couples pool resources to maximize utility in the presence of increased transaction costs.

#### 5.2.3 Relationship history and duration

Results from the third set of models in Table 2 indicate that, as hypothesised, relationship history and duration are significant predictors of banking arrangements. In the random-effect logit model, remarried/re-partnered couples have much lower odds of having joint accounts than couples in their first marriages/*de facto* relationships (OR from 0.04 to 0.32, *p<*0.001). In the random-effect multinomial logit model, we further learn that remarriage/re-partnership raises the odds of having separate accounts for either or both partners. In a similar vein, relationship duration is positively associated with the odds of having a joint account in the random-effect logit model (OR = 1.07, *p<*0.001), and negatively associated with the odds of all arrangements involving separate accounts in the random-effect multinomial logit model (OR_men_sep_ = 0.98, *p*<0.05; OR_wom_sep_ = 0.97, OR_both_sep+joint_ = 0.95, OR_both_sep_ = 0.94, *p<*0.001).

#### 5.2.4 Gender-role attitudes

The fourth set of models in Table 2 considers the relationships between gender ideology and banking arrangements. Traditional gender attitudes are not associated with the odds of couples having joint accounts in the random-effect logit model. However, results in the more telling random-effect multinomial logit model indicate that such attitudes are associated with reduced odds of both partners having separate accounts (OR_both_sep+joint_ = 0.99, *p<*0.001; OR_both_sep_ = 0.99, *p*<0.05). This is consistent with our fourth hypothesis.

Following a suggestion by an anonymous reviewer, we also considered the role of within-couple differences in gender-role attitudes. To accomplish this, we constructed a categorical variable comparing the male and female partner scores in the gender-attitude index. This new variable distinguished couples in which: (i) the male partner held more traditional attitudes than the female partner, (ii) both partners had the same attitudinal score (reference category), and (iii) the female partner held more traditional attitudes than the male partner. In these models, we also controlled for the average couple-level score in the gender-attitudes index used in the main models. The coefficients on the latter were highly consistent with those presented in Table 2. The coefficients on the categories of the new within-couple difference variable were generally statistically insignificant. There were however some exceptions. Relative to couples in which both partners held the same attitudes, in couples in which the male partner held more traditional attitudes (i.e., the female partner held more progressive attitudes) both partners (OR = 1.28, p<0.05) and the female partner (OR = 1.33, p<0.05) had higher odds of having separate accounts. This suggests that, consistent with previous studies [9,27,45], women are more likely to have separate accounts if they hold more egalitarian attitudes towards gender roles than their male partners.

#### 5.2.5 Intergenerational effects

The final set of models in Table 2 examines the associations between parental characteristics and couples’ banking arrangements. In the random-effect logit model, couples in which both partners come from female-empowered family backgrounds have much lower odds of having joint accounts (OR = 0.56, *p<*0.05). The random-effect multinomial logit model suggests that couples from higher SES family backgrounds have higher odds of having a mixed banking portfolio involving joint and separate accounts for both partners (OR_both_sep+joint_ = 1.01, *p<*0.05). Couples in which both partners come from female-empowered family backgrounds have significantly higher odds of having separate accounts only (OR_both sep_ = 1.67, *p*<0.05). These findings partially support our final hypothesis.

#### 5.2.6 The predictive role of other socio-demographic factors

S1–S6 Tables show the predicted effects of the control variables on couples’ banking arrangements. While examining these is not the key contribution of the present study, doing so generates additional theoretically-relevant insights. It also sheds light on whether socio-demographic factors deemed as important predictors in previous international studies hold that status within the contemporary Australian context.

Consistent with previous research, the odds of having joint bank accounts are generally significantly higher and the odds of having separate bank accounts significantly lower when couples are older, have smaller age gaps, and are married [1,14,16]. Also consistent with expectations [33,38,50], couples with higher socio-economic status (measured by employment and education) are generally significantly more likely to hold separate bank accounts together with a joint account.

Concerning ethno-migrant background, there is some evidence that culturally and linguistically diverse communities in Australia diverge to some extent from Anglo-Celtic practices: couples with at least one Australian-born partner are significantly more likely to have joint bank accounts than other couples. This aligns with the proposition that joint accounts in marriage are less common in the global South [2,5].

### 5.3 Do married and cohabiting couples handle money differently?

Married couples and those living in *de facto* relationships are different in a number of ways (e.g. religiosity, education or gender attitudes), with differences extending to choices relating to money control and money management [1,7,16,61]. Compared to marriages, cohabitating relationships involve fewer norms, obligations and formal ties, and as a result more independence [1]. As a result, cohabiting couples often opt for holding money separately as a reflection of ‘equality’, even if this may not always result in more ‘equity’ in these relationships [5,38,62]. Consistent with these arguments, cohabiting couples in the US and Sweden [16] and Norway [1] are much more likely than married couples to keep money separate, and a greater share of Australian married couples than cohabiting couples have joint accounts [33].

In this section we outline the results of additional analyses in which we split the overall sample into sub-samples of married and cohabiting couples. We not only consider differences in the prevalence of different banking arrangements between cohabiting and married couples, but also add to knowledge by exploring whether the factors predicting these choices differ by relationship type.

**Fig 2:**
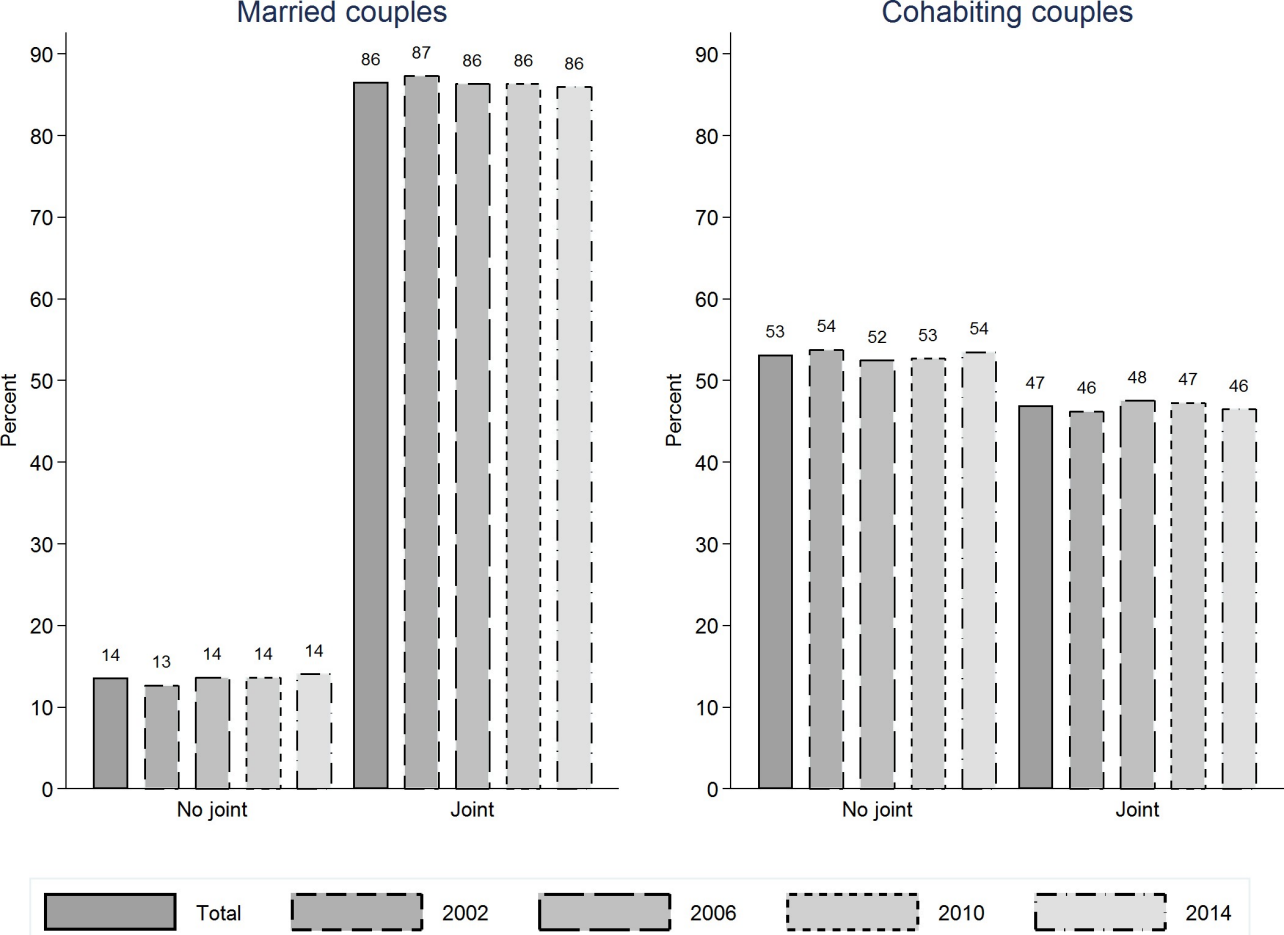
Trends over time in the banking arrangements of heterosexual couples in Australia, comparison of married vs. cohabiting couples (binary measure).

**Fig 3:**
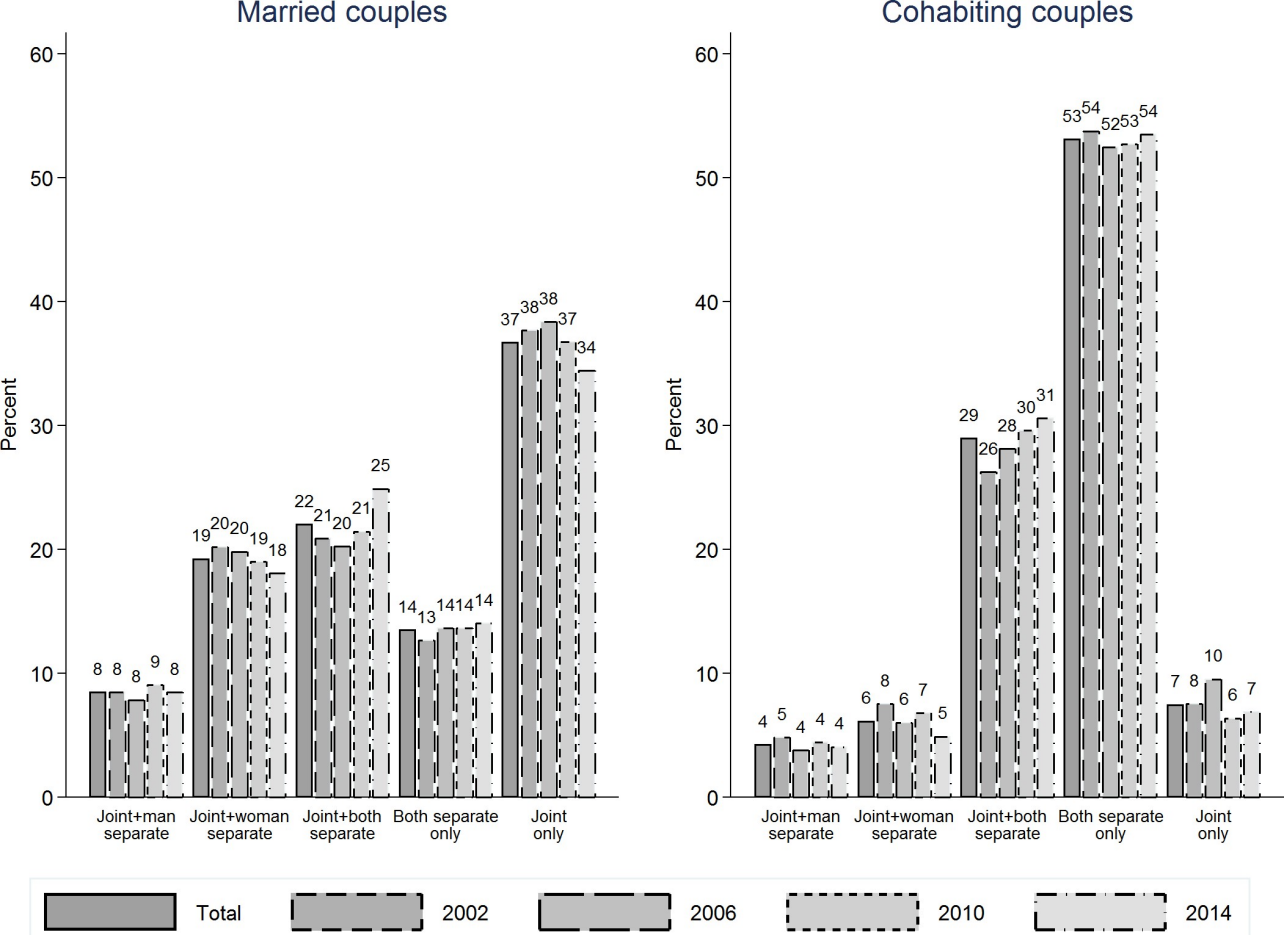
Trends over time in the banking arrangements of heterosexual couples in Australia, comparison of married vs. cohabiting couples (multinomial measure).

Results in Fig 2 are for the binary measure of banking arrangements capturing whether couples have a joint account, and are consistent with cross-sectional reports in Singh and Morley [33]. While 86% of married couples have joint accounts, only 47% of cohabiting couples do so. Further, there is no evidence of change over time in this difference. Results in Fig 3 are for the more detailed five-group measure of banking arrangements, and reveal that over time trends in the prevalence of different financial arrangements have been consistent for married and cohabiting couples. In other words, trends are universal and these two types of unions are not increasingly diverging from each other.

HILDA Survey (2002, 2006, 2010 & 2014). Married couples (left panel): n(observations): 12,518; n(couples): 5,480. Cohabiting couples (right panel): n(observations): 2,861; n(couples): 2,036.

HILDA Survey (2002, 2006, 2010 & 2014). Married couples (left panel): n(observations): 12,518; n(couples): 5,480. Cohabiting couples (right panel): n(observations): 2,861; n(couples): 2,036.

Table 3 shows the results of random-effect logistic regression models estimated separately for the sub-samples of married and cohabiting couples. Generally, these provide evidence of similarity rather than difference in the ways in which our key explanatory variables relate to banking arrangements across these sub-samples. As an exception, there are some significant differences concerning relationship history and duration. Of note, relationship duration is more predictive of joint banking in cohabiting than married couples. This effect likely signifies the importance of union duration as a *proxy* for mutual commitment to a shared future in *de facto* relationships. It is consistent with the *relationship duration effect* discussed by Lyngstad, Noack and Tufte [1], and with their findings that cohabiting couples intending to marry were more likely to pool resources. Couples move towards ‘jointness’ as they transition to marriage, but also as they consolidate as long-term committed cohabiting unions.

**Table 3:** Banking arrangements among heterosexual couples in Australia, comparing married and cohabiting couples.

	Joint account vs. no joint account			
	Cohabiting couples	Married couples	Wald test (*p*)	Full sample
*Hypothesis 1* ^a^				
Total income (IHS)	1.70^**^	1.18^*^	0.059	1.31^***^
Relative resources (ref. similar contribution)				
Women contribute 60%+	0.70	0.73	0.893	0.74^*^
Men contribute 60%+	0.92	1.15	0.280	1.06
*Hypothesis 2* ^b^				
Number of dependent children	1.28^**^	1.34^***^	0.673	1.32^***^
*Hypothesis 3* ^c^				
Relationship history (ref. both 1^st^ relationship)				
Men 1^st^ relationship and women 2^nd^+	3.55	0.60	0.033^*^	0.22^***^
Women 1^st^ relationship and men 2^nd^+	2.58	1.07	0.295	0.32^***^
Both 2^nd^+ relationship	3.80^*^	1.31	0.145	0.04^***^
Relationship duration	1.21^***^	1.14^***^	0.023^*^	1.07^***^
*Hypothesis 4* ^b^				
Gender-role attitudes	0.99	1.00	0.285	1.00
*Hypothesis 5* ^b^				
Mean parental socio-economic status	1.00	1.01	0.194	1.00
Family background (ref. neither from female-empowered family)				
Only man from female-empowered family	0.63	0.80	0.577	0.66
Only woman from female-empowered family	1.01	0.75	0.469	0.81
Both from female-empowered family	0.57	0.61^*^	0.889	0.56^*^

HILDA Survey (2002, 2006, 2010 & 2014). Odds ratios from random-effect binary logit models. Wald tests compare the coefficients in the model for married couples and the model for cohabiting couples. Samples sizes range from 11,645 to 12,518 observations (5,140 to 5,480 couples) for married couples, and from 2,436 to 2,861 observations (1,728 to 2,036 couples) for cohabiting couples. All models feature robust standard errors.

^a^ controls: marital status, age, employment, education and ethnicity.

^b^ controls: marital status, age, employment, education, ethnicity and total income (IHS).

^c^ controls: age, employment, education, ethnicity and total income.

^*^ *p<*0.05,

^**^ *p<*0.01,

^***^ *p<*0.001. Complete tables of model coefficients are available from the authors upon request.

### 5.4 Additional specifications and robustness checks

To complement the main models presented thus far, we undertook a series of sensitivity analyses aimed at testing the robustness of our results to different model specifications and analytical decisions. We are thankful to several anonymous reviewers for suggesting these. In this section, we report on these additional results.

First, we repeated our analyses excluding couples who were part of the top-up sample added to the HILDA Survey in 2011 (1,088 couples, all with one observation). Because these couples only participated in one of the survey waves used in the present study (Wave 14 in 2014), it is possible that their inclusion could have distorted our results. Reassuringly, the latter was not the case. Changes in the prevalence of different bank accounts in 2014 for this revised sample (not shown but available upon request) were in the second decimal. Further, as can be appreciated by inspection of S7 Table, changes in the magnitude and statistical significance of the odds ratios (relative to those from the main models reported in Table 2) were minimal. The few odds ratios which lost statistical significance (e.g., the odds ratio for relative income in the logit model) were similar in magnitude to those in the main models. This suggests that the loss of statistical significance is the result of a smaller sample size.

Second, we used a different strategy to deal with couples in which the male and female partner did not agree on whether they had joint bank accounts. In the main models (Table 2), we coded those cases as if the couple did have a joint bank account. In the new specification (S8 Table), we coded those cases as if the couple did not have a joint bank account. This recoding resulted in a sample loss of 1,704 observations from 1,122 couples. The regression results were however highly consistent, with only minor changes in the magnitude, direction and statistical significance of the odds ratios.

Third, our main results come from random-effect (binary and multinomial) panel regression models. However, fixed-effect models could be perceived as an alternative estimation approach. The usual tradeoff between these approaches is one between unbiasedness (favoring fixed-effect models) and efficiency (favoring random-effect modelling). In this occasion, we opted for random-effect over fixed-effect models for two key reasons. First, with our data structure, some of our key explanatory variables are time-constant (e.g., parental background and relationship history). Fixed-effect models are unable to accommodate time-constant regressors, and hence could not be deployed to examine how these factors were associated with bank account choices [63]. Second, many couples experience no change over time in their bank account choices, our outcome variable, over the observation window. Specifically, change is never observed for 90.1% of all couples for the binary outcome, and 68.8% for the multinomial outcome. This is critical in shifting the balance in favor of random-effect models, as fixed-effect models are estimated using only information from those couples for which change over time in the outcome variable is observed [63]. As a result, modelling these data using fixed-effect rather than random-effect models incurs a sample loss of 13,265 observations from 6,355 couples for the binary outcome and 8,126 observations from 4,855 couples for the multinomial outcome—that is, about 86.3% of observations and 90.1% of couples for the binary outcome, and 52.8% of observations and 68.8% of couples for the multinomial outcome. The resulting fixed-effect models are therefore selective towards couples which experience change, and highly inefficient. For interested readers, results based on fixed-effect models for the time-varying explanatory variables are presented in S9 Table. As suspected, the odds ratios in these models are rarely statistically significant—although their magnitude and sign are generally similar to those in the random-effect models, reflecting the very substantial efficiency loss incurred by fixed-effect models. Researchers sometimes use the Hausman specification test [64] to choose between random-effect and fixed-effect models. Given the large differences between the random-effect and fixed-effect coefficients in our logistic model (see Tables 2 and S9), it is unsurprising that a Hausman test rejects the null hypothesis that the two sets of coefficients are not systematically different (p < 0.001). While this would usually be taken as evidence in favor of fixed-effect models, for the two critical reasons outlined in the body of the text, fixed-effect models are not feasible or sufficiently efficient with our data. There is no Hausman-test equivalent for multinomial specifications, and so we cannot undertake similar comparisons for models in which the outcome is the 5-category measure of bank account choices.

## 6 Discussion and conclusion

In this paper we have examined the prevalence of different banking arrangements among heterosexual couples in contemporary Australia, as well as changes over time. We also paid attention to how economic, life course, socio-cultural, and intergenerational factors predicted these arrangements. In doing so, we provided a more encompassing and granular picture of within-couple banking arrangements than ever before. Our empirical analyses were undertaken using a large, nationally representative household panel survey comprising the period 2002–2014, exploiting both its panel structure (by estimating state-of-the-art panel regression models for the first time in this field) and its household structure (by leveraging couple-level data that better reflects partnership circumstances and improves estimation).

Findings indicate that in contemporary Australia the majority of couples (79%) have a joint bank account, consistent with recent research in the US [13], the UK [14], Canada [45], as well as earlier Australian research [33]. Hence, the joint bank account remains an important element in the financial organization of Australian households—one that helps negotiate what Singh referred to as the central dilemma of modern life in Western countries [2]: how to manage committed personal and family relationships on the one hand and women’s progressive financial independence on the other. Some scholars have suggested that changes towards financial separateness in couples’ banking arrangements both reflect and contribute to processes of modernization and individualization [12,32]. Our trend analyses provided some evidence of shifts towards financial autonomy and separateness in Australian families. For instance, there was a moderate decrease over time in the share of couples holding joint accounts (81% in 2002 compared to 78% in 2014). There was also a decrease in the share of couples holding only joint accounts (from 33% to 29%), and increases in the share of couples in which both partners had separate accounts in addition to joint accounts (from 22% to 26%) or separate accounts only (from 19% to 22%). This pattern of results is largely consistent with recent research using UK longitudinal data by Kan and Laurie [14], who reported increasing independence in financial arrangements within couples over the 1995–2005 period.

Concerning their predictors, we provided novel evidence that the banking arrangements of heterosexual couples in contemporary Australia are associated with previously untested (or rarely tested) economic, life-course, socio-cultural and intergenerational factors. Altogether, we found robust evidence supporting Hypotheses 1 to 3 (relative resources, transaction costs, relationship history and duration), and some evidence in support of Hypotheses 4 and 5 (gender ideology, intergenerational factors). Economic factors were found to be important predictors of couples’ banking arrangements. Both absolute and relative income predicted these in theoretically expected ways: high absolute income was associated with increased odds of joint account ownership, and so were comparable income contributions to the household by couple members [3,17]. This pattern of results concerning spousal bargaining power is also consistent with findings from other literatures examining couple-level outcomes (e.g., labor supply and housework divisions). Interestingly, women’s contributions to total couple income were more predictive of separate bank accounts than men’s. In other words, women’s economic resources are particularly important contributors to financial independence. Our results were also consistent with the hypothesis that the number of children in the household would predict increased odds of joint bank account ownership and decreased odds of banking arrangements involving separate accounts. We take this finding as suggestive evidence of the notion of ‘transaction costs’ [7], i.e., couples opting for a joint banking strategy as a means to minimize negotiations and disputes on the source of payments associated with their collective capitals.

Life-course factors were also precursors of couples’ banking arrangements. Particularly, shorter and more complicated relationship histories were associated with couples more often relying on separate accounts. This finding highlights the importance of considering banking arrangements within a life course perspective: past experiences have the potential to alter current day-to-day family life arrangements, and different individual and couple life-course stages are associated with diverging practices [1,13,37,48]. Further, socio-cultural aspects, measured through individual attitudes, were also predictive of banking arrangements in theoretically meaningful ways. We show that traditional gender-role attitudes are negatively related to the odds of couple members using separate banking, though only weakly. This constitutes novel evidence that attitudes are important contributors to individuals’ banking arrangements net of material/tangible factors, and evidence of couples ‘doing gender’ when making banking decisions. This finding adds to a body of knowledge documenting the associations between gender-role attitudes and individuals’ behaviors across life domains, e.g., labor, childcare and housework supply, union formation and dissolution, leisure time allocations, or marital conflict [49].

Finally, we provided first-time quantitative evidence of intergenerational associations between parental characteristics and adult children’s banking arrangements. Specifically, we found that parental SES is significantly and positively associated with the odds of both partners holding joint and separate accounts, and that ‘female-empowered’ family backgrounds are associated with an increased prevalence of separate banking arrangements. This resonates with arguments in the literature that, to gain a better understanding of family banking practices, we need to pay attention to the wider family and examine cross-generational relationships [5].

As in previous studies [14,33], there were striking differences between couple types in the prevalence of joint accounts: 86% amongst married couples compared to 47% amongst cohabiting couples. This is consistent with the notion that individuals who decide to marry are different in their financial beliefs and practices to those who opt for cohabitation [16,24, 59]. Interestingly, the observed trends towards financial separateness went in the same direction and were of a similar magnitude amongst married and cohabiting couples alike. This suggests that the aforementioned processes of modernization and individualization in families and partnerships, which have been considered chiefly in relation to marriage [12,32], are also in motion amongst cohabiting couples. This means that two reinforcing factors are at play in shifting the financial practices of Australian families towards separateness: the growth of cohabiting couples (which are less likely to pool resources) and a trend over time towards separateness amongst both married and cohabiting unions. The universality of these processes deserves further scholastic attention.

Our results speak of similarities and differences in the predictors of different bank account arrangements between Australia and other countries. Similar to studies in the US, the UK and Norway, we find that egalitarian contributions to household income, dependent children and longer relationships are all positively associated with joint account ownership and negatively associated with separate accounts. However, our finding of a negative association between traditional gender ideology and couples’ ownership of separate bank accounts in Australia is at odds with findings from a US study [16] reporting no such association. This could reflect Australia’s unique historical legacy: institutional inertia due to previous legislation reinforcing the male-breadwinner model may still influence the behaviors and outcomes of couples in contemporary Australia. More broadly, our study has added Australia as a comparison benchmark to existing evidence for the US, the UK, Canada, Norway and South Korea. Pooling the results from our study and these other studies, we now have a relatively good understanding of the micro-level factors associated with different bank account arrangements within couples. Yet, we have virtually no evidence on the role of macro-level factors in influencing couples’ bank account arrangements. One set of such macro-level factors may operate through institutional environments, as hinted by country-level differences in the prevalence of joint bank accounts across studies—with the United States (very prevalent) and South Korea (virtually inexistent) as two extremes, and Australia, the UK and Canada falling in-between. Hence, a promising research avenue within this field of enquiry would be to systematically examine the country-level factors associated with within-couple banking arrangements [3,12].

What do these results mean for gender inequality? As explained before, it has been argued that jointness in financial management leads to better comparative outcomes for partnered women [3,9,15,44]. We found a rather strong patterning of bank account choices by several socio-demographic factors. This suggests that certain ‘types’ of women may benefit more than others from the benefits associated with financial jointness reported in other studies [9,13–15,37–39]. In addition, the observed time trends towards financial separateness suggest that fewer women are—and will be—benefiting from any advantages associated with resource pooling. An alternative take on this is that partnered women are progressively steering towards ‘equality’ (more equal financial behaviors and practices to those their male partners) than ‘equity’ (more equal access to money and outcomes). In light of persistent gender inequalities at work, it is also possible that the women moving away from financial jointness are those women who are financially better-off relative to their partners. This is consistent with our results showing that women who contribute a higher share of income to the household are more likely to lean towards financial autonomy via separate accounts. But this reality applies to only a small proportion of partnered Australian women, with male partners being the main breadwinners in 70% of Australian couple households in our data. Our findings also confirm that factors known to produce gender differences in behaviors and outcomes in other domains (e.g., employment and housework) also produce gendered behaviors and outcomes in relation to couples’ banking arrangements. These include income, gender ideology and partnership history. A relatively surprising finding was that couples in which only the female partner had a separate bank account outnumbered those in which only the male partner did so. This finding suggests a greater tendency for women to ‘hold money back’ from the male partners than *vice versa*, and is consistent with cross-sectional findings in Singh and Morley [33], as well as early qualitative studies such as Pahl [65] and Laurie [66]. Female intra-household money management may not reflect female control over money, but rather executive responsibility in undertaking gendered household tasks—e.g., grocery shopping or paying bills [3,44]. That is, our results may reflect the tendency for women to take responsibility for household payments as part of their domestic roles, for which a separate account may be more convenient and less disruptive to the male partner [67].

Despite our several contributions to the scant quantitative literature on banking arrangements, our study has some limitations which could be addressed in future research. First, our operationalization of certain explanatory variables is occasionally hampered by data quality. Particularly, our gender-attitude measure is not concurrent and neglects the fact that such attitudes can change [68], and our parental background variables are retrospectively reported by adult children, which may lead to measurement error [69]. Additionally, we lacked information on parents’ bank account choices, financial arrangements, gender divisions and gender ideology, all of which would have added depth to our intergenerational analyses. Second, our analyses are based on unique and rich data on the type and number of bank accounts held by couple members, which allowed us to improve upon most of the earlier studies. Yet, importantly, we do not have information on relevant contextual factors that could yield further insights. For example, we lack information on how much money is held into each joint and separate account, the share of the money contributed by each partner, and the specific source of that money (e.g., regular labor income, over time earnings, welfare benefits, gifts, etc.). Access to such information would enable to better quantify the actual amount of money available to or controlled by each partner. Relatedly, even when money is held in a joint account, this does not mean that both partners think of or use such money in the same ways. Qualitative studies have documented differences by gender and breadwinner status in aspects such as the perceived ownership of money, the uses deemed appropriate for different monies, or the amount of consumption and personal spending [2,8,47]. Routine inclusion of questions on these matters in large-scale social and economic surveys would enable more nuanced quantitative analyses of these processes that would advance the field.

## Supporting information

S1 Table Banking arrangements among heterosexual couples in Australia, full output for the baseline model. HILDA Survey (2002, 2006, 2010 & 2014). Odds ratios. All models feature robust standard errors. ^*^ *p<*0.05, ^**^ *p<*0.01, ^***^ *p<*0.001.
(DOCX)

S2 Table Banking arrangements among heterosexual couples in Australia, full output for models testing Hypothesis 1. HILDA Survey (2002, 2006, 2010 & 2014). Odds ratios. All models feature robust standard errors. ^*^ *p<*0.05, ^**^ *p<*0.01, ^***^ *p<*0.001.
(DOCX)

S3 Table Banking arrangements among heterosexual couples in Australia, full output for models testing Hypothesis 2. HILDA Survey (2002, 2006, 2010 & 2014). Odds ratios. All models feature robust standard errors. ^*^ *p<*0.05, ^**^ *p<*0.01, ^***^ *p<*0.001.
(DOCX)

S4 Table Banking arrangements among heterosexual couples in Australia, full output for models testing Hypothesis 3. HILDA Survey (2002, 2006, 2010 & 2014). Odds ratios. All models feature robust standard errors. ^*^ *p<*0.05, ^**^ *p<*0.01, ^***^ *p<*0.001.
(DOCX)

S5 Table Banking arrangements among heterosexual couples in Australia, full output for models testing Hypothesis 4. HILDA Survey (2002, 2006, 2010 & 2014). Odds ratios. All models feature robust standard errors. ^*^ *p<*0.05, ^**^ *p<*0.01, ^***^ *p<*0.001.
(DOCX)

S6 Table S6Banking arrangements among heterosexual couples in Australia, full output for models testing Hypothesis 5. HILDA Survey (2002, 2006, 2010 & 2014). Odds ratios. All models feature robust standard errors. ^*^ *p<*0.05, ^**^ *p<*0.01, ^***^ *p<*0.001.
(DOCX)

S7 Table Banking arrangements among heterosexual couples in Australia, HILDA Survey top-up sample excluded. HILDA Survey (2002, 2006, 2010 & 2014). Column 1: random-effect binary logit models. Columns 2–4: random-effect multinomial logit models. All models feature robust standard errors. ^a^ controls: marital status, age, employment, education and ethnicity. ^b^ controls: marital status, age, employment, education, ethnicity and total income (IHS). ^c^ controls: age, employment, education, ethnicity and total income. ^*^ *p<*0.05, ^**^ *p<*0.01, ^***^ *p<*0.001. Complete tables of model coefficients are available from the authors upon request.
(DOCX)

S8 Table Banking arrangements among heterosexual couples in Australia, models where couple-level mismatches in joint bank accounts are coded as ‘no joint account’. HILDA Survey (2002, 2006, 2010 & 2014). Column 1: random-effect binary logit models. Columns 2–4: random-effect multinomial logit models. All models feature robust standard errors. ^a^ controls: marital status, age, employment, education and ethnicity. ^b^ controls: marital status, age, employment, education, ethnicity and total income (IHS). ^c^ controls: age, employment, education, ethnicity and total income. ^*^ *p<*0.05, ^**^ *p<*0.01, ^***^ *p<*0.001. Complete tables of model coefficients are available from the authors upon request.
(DOCX)

S9 Table Banking arrangements among heterosexual couples in Australia, fixed-effect specifications. HILDA Survey (2002, 2006, 2010 & 2014). Column 1: fixed-effect binary logit models. Columns 2–4: fixed-effect multinomial logit models. Couples’ age difference and born in Australia are time constant and dropped in the models.
(DOCX)
