# Supplementary material for: To pool or not to pool? Trends and predictors of banking arrangements within Australian couples
Source: PLoS One. 2019 Apr 17;14(4):e0214019. doi: 10.1371/journal.pone.0214019 (PMC6469846; doi:10.1371/journal.pone.0214019)
Supplement: S1 Table — HILDA Survey (2002, 2006, 2010 & 2014). Odds ratios. All models feature robust standard errors. * p<0.05, ** p<0.01, *** p<0.001. (DOCX) [file pone.0214019.s001.docx]

**Table S1. Banking arrangements among heterosexual couples in Australia, full output for the baseline model.**

|  | Joint account  vs. no joint  account | Account choice (ref. partners have only a joint account) | | | |
| --- | --- | --- | --- | --- | --- |
|  |  | Joint + man  separate | Joint + woman  separate | Joint + both  separate | Both separate only |
| Couple’s mean age | 1.06^***^ | 0.97^***^ | 0.99^*^ | 0.98^***^ | 0.96^***^ |
| Couples’ age difference (<=5 years) |  |  |  |  |  |
| Man 5 years older | 0.42^***^ | 1.44^*^ | 1.17 | 1.47^**^ | 1.88^***^ |
| Woman 5 years older | 0.30^***^ | 1.44 | 1.76 | 2.42^**^ | 3.16^***^ |
| Marital status (*de facto*) |  |  |  |  |  |
| Married | 64.13^***^ | 0.11^***^ | 0.15^***^ | 0.04^***^ | 0.02^***^ |
| Employment status (neither employed) |  |  |  |  |  |
| Both employed | 3.21^***^ | 1.19 | 1.76^***^ | 1.25 | 0.63^**^ |
| Only man employed | 2.52^***^ | 1.57^*^ | 1.71^***^ | 1.08 | 0.78 |
| Only woman employed | 1.11 | 1.68^+^ | 2.25^***^ | 1.88^**^ | 1.69^*^ |
| University degree (neither has degree) |  |  |  |  |  |
| Both have degrees | 1.63^**^ | 1.92^***^ | 1.33^+^ | 1.87^***^ | 1.45^*^ |
| Only man has a degree | 1.43 | 1.43^+^ | 1.15 | 1.58^**^ | 1.20 |
| Only woman has a degree | 1.30 | 1.32 | 0.92 | 1.19 | 1.04 |
| Born in Australia (neither) ^a^ |  |  |  |  |  |
| Both born in Australia | 1.56^**^ | 1.14 | 1.60^***^ | 1.63^***^ | 1.14 |
| Only man born in Australia | 1.93^*^ | 1.80^*^ | 1.98^**^ | 2.50^***^ | 1.42 |
| Only woman born in Australia | 1.71^*^ | 1.28 | 1.52^*^ | 1.75^**^ | 1.11 |
| Couple’s total income (IHS) | 1.32^***^ | 1.29^***^ | 1.09 | 1.28^***^ | 0.94 |
| N (observations) | 15,379 | 15,379 | | | |
| N (couples) | 7,054 | 7,054 | | | |
| AIC/BIC | 11,159/11,281 | 40,960/41,426 | | | |

HILDA Survey (2002, 2006, 2010 & 2014). Odds ratios. All models feature robust standard errors. ^*^ *p<*0.05, ^**^ *p<*0.01, ^***^ *p<*0.001.
