# Supplementary material for: To pool or not to pool? Trends and predictors of banking arrangements within Australian couples
Source: PLoS One. 2019 Apr 17;14(4):e0214019. doi: 10.1371/journal.pone.0214019 (PMC6469846; doi:10.1371/journal.pone.0214019)
Supplement: S2 Table — HILDA Survey (2002, 2006, 2010 & 2014). Odds ratios. All models feature robust standard errors. * p<0.05, ** p<0.01, *** p<0.001. (DOCX) [file pone.0214019.s002.docx]

**Table S2. Banking arrangements among heterosexual couples in Australia, full output for models testing Hypothesis 1.**

|  | Joint account  vs. no joint  account | Banking arrangements (ref. partners have only a joint account) | | | |
| --- | --- | --- | --- | --- | --- |
|  |  | Joint+man separate | Joint+woman  separate | Joint+both  separate | Both separate only |
| Couple’s mean age | 1.06^***^ | 0.97^***^ | 0.99^*^ | 0.98^***^ | 0.96^***^ |
| Couples’ age difference (<=5 years) |  |  |  |  |  |
| Man 5 years older | 0.42^***^ | 1.43^*^ | 1.17 | 1.46^**^ | 1.86^***^ |
| Woman 5 years older | 0.31^***^ | 1.43 | 1.75 | 2.40^**^ | 3.13^***^ |
| Marital status (*de facto*) |  |  |  |  |  |
| Married | 63.26^***^ | 0.11^***^ | 0.15^***^ | 0.04^***^ | 0.02^***^ |
| Employment status (neither employed) |  |  |  |  |  |
| Both employed | 3.22^***^ | 1.17 | 1.68^***^ | 1.23 | 0.62^**^ |
| Only man employed | 2.45^***^ | 1.52^*^ | 1.57^**^ | 1.09 | 0.77 |
| Only woman employed | 1.23 | 1.57 | 1.98^**^ | 1.67^*^ | 1.45 |
| University degree (neither has degree) |  |  |  |  |  |
| Both have degrees | 1.65^**^ | 1.91^***^ | 1.31 | 1.85^***^ | 1.44^*^ |
| Only man has a degree | 1.42 | 1.41 | 1.12 | 1.57^**^ | 1.19 |
| Only woman has a degree | 1.36 | 1.30 | 0.91 | 1.15 | 1.00 |
| Born in Australia (neither) ^a^ |  |  |  |  |  |
| Both born in Australia | 1.55^*^ | 1.13 | 1.58^**^ | 1.63^***^ | 1.14 |
| Only man born in Australia | 1.92^*^ | 1.77^*^ | 1.92^**^ | 2.48^***^ | 1.40 |
| Only woman born in Australia | 1.73^*^ | 1.27 | 1.50^*^ | 1.73^**^ | 1.09 |
| Total income (IHS) | 1.31^***^ | 1.29^***^ | 1.08 | 1.30^***^ | 0.94 |
| Relative resources (ref. similar contribution) |  |  |  |  |  |
| Women contribute 60%+ | 0.74^*^ | 1.30 | 1.59^***^ | 1.39^**^ | 1.59^***^ |
| Men contribute 60%+ | 1.06 | 1.11 | 1.29^**^ | 1.00 | 1.07 |
| N (observations) | 15,379 | 15,379 | | | |
| N (couples) | 7,054 | 7,054 | | | |
| AIC/BIC | 11,158/11,295 | 40,944/41,471 | | | |

HILDA Survey (2002, 2006, 2010 & 2014). Odds ratios. All models feature robust standard errors. ^*^ *p<*0.05, ^**^ *p<*0.01, ^***^ *p<*0.001.
