# Supplementary material for: To pool or not to pool? Trends and predictors of banking arrangements within Australian couples
Source: PLoS One. 2019 Apr 17;14(4):e0214019. doi: 10.1371/journal.pone.0214019 (PMC6469846; doi:10.1371/journal.pone.0214019)
Supplement: S3 Table — HILDA Survey (2002, 2006, 2010 & 2014). Odds ratios. All models feature robust standard errors. * p<0.05, ** p<0.01, *** p<0.001. (DOCX) [file pone.0214019.s003.docx]

**Table S3. Banking arrangements among heterosexual couples in Australia, full output for models testing Hypothesis 2.**

|  | Joint account  vs. no joint  account | Banking arrangements (ref. partners have only a joint account) | | | |
| --- | --- | --- | --- | --- | --- |
|  |  | Joint+man separate | Joint+woman  separate | Joint+both  separate | Both separate only |
| Couple’s mean age | 1.06^***^ | 0.97^***^ | 0.99^*^ | 0.97^***^ | 0.95^***^ |
| Couples’ age difference (<=5 years) |  |  |  |  |  |
| Man 5 years older | 0.39^***^ | 1.47^**^ | 1.19 | 1.55^**^ | 1.98^***^ |
| Woman 5 years older | 0.30^***^ | 1.44 | 1.77 | 2.38^**^ | 3.11^***^ |
| Marital status (*de facto*) |  |  |  |  |  |
| Married | 54.50^***^ | 0.12^***^ | 0.16^***^ | 0.05^***^ | 0.02^***^ |
| Employment status (neither employed) |  |  |  |  |  |
| Both employed | 3.21^***^ | 1.20 | 1.77^***^ | 1.27 | 0.64^**^ |
| Only man employed | 2.29^***^ | 1.60^**^ | 1.74^***^ | 1.17 | 0.85 |
| Only woman employed | 1.09 | 1.68 | 2.28^***^ | 1.87^**^ | 1.70^*^ |
| University degree (neither has degree) |  |  |  |  |  |
| Both have degrees | 1.71^**^ | 1.95^***^ | 1.34^*^ | 1.88^***^ | 1.45^*^ |
| Only man has a degree | 1.48 | 1.44^*^ | 1.16 | 1.58^**^ | 1.19 |
| Only woman has a degree | 1.35 | 1.31 | 0.91 | 1.18 | 1.02 |
| Born in Australia (neither) ^a^ |  |  |  |  |  |
| Both born in Australia | 1.56^**^ | 1.15 | 1.62^***^ | 1.64^***^ | 1.15 |
| Only man born in Australia | 1.96^**^ | 1.81^*^ | 2.00^**^ | 2.49^***^ | 1.41 |
| Only woman born in Australia | 1.75^*^ | 1.28 | 1.53^*^ | 1.75^**^ | 1.10 |
| Total income (IHS) | 1.28^***^ | 1.29^***^ | 1.09 | 1.31^***^ | 0.96 |
| Number of dependent children | 1.32^***^ | 0.88^**^ | 0.92 | 0.77^***^ | 0.76^***^ |
| N (observations) | 15,379 | 15,379 | | | |
| N (couples) | 7,054 | 7,054 | | | |
| AIC/BIC | 11,130/11,260 | 40,872/41,369 | | | |

HILDA Survey (2002, 2006, 2010 & 2014). Odds ratios. All models feature robust standard errors. ^*^ *p<*0.05, ^**^ *p<*0.01, ^***^ *p<*0.001.
