# Supplementary material for: To pool or not to pool? Trends and predictors of banking arrangements within Australian couples
Source: PLoS One. 2019 Apr 17;14(4):e0214019. doi: 10.1371/journal.pone.0214019 (PMC6469846; doi:10.1371/journal.pone.0214019)
Supplement: S4 Table — HILDA Survey (2002, 2006, 2010 & 2014). Odds ratios. All models feature robust standard errors. * p<0.05, ** p<0.01, *** p<0.001. (DOCX) [file pone.0214019.s004.docx]

**Table S4. Banking arrangements among heterosexual couples in Australia, full output for models testing Hypothesis 3.**

|  | Joint account  vs. no joint  account | Banking arrangements (ref. partners have only a joint account) | | | |
| --- | --- | --- | --- | --- | --- |
|  |  | Joint+man separate | Joint+woman  separate | Joint+both  separate | Both separate only |
| Couple’s mean age | 1.03^***^ | 0.98 | 1.01 | 1.00 | 0.99 |
| Couples’ age difference (<=5 years) |  |  |  |  |  |
| Man 5 years older | 0.75 | 1.08 | 0.88 | 0.96 | 1.08 |
| Woman 5 years older | 0.61 | 0.98 | 1.05 | 1.29 | 1.47 |
| Employment status (neither employed) |  |  |  |  |  |
| Both employed | 4.46^***^ | 1.14 | 1.68^***^ | 1.05 | 0.47^***^ |
| Only man employed | 3.70^***^ | 1.49^*^ | 1.62^**^ | 0.89 | 0.55^***^ |
| Only woman employed | 1.42 | 1.69 | 2.24^***^ | 1.72^*^ | 1.41 |
| University degree (neither has degree) |  |  |  |  |  |
| Both have degrees | 1.77^**^ | 1.87^***^ | 1.29 | 1.73^***^ | 1.35 |
| Only man has a degree | 1.72^*^ | 1.33 | 1.10 | 1.42^*^ | 1.03 |
| Only woman has a degree | 1.46^*^ | 1.24 | 0.85 | 1.05 | 0.91 |
| Born in Australia (neither) ^a^ |  |  |  |  |  |
| Both born in Australia | 1.57^*^ | 1.14 | 1.61^***^ | 1.65^***^ | 1.19 |
| Only man born in Australia | 2.81^***^ | 1.53 | 1.65^*^ | 1.91^**^ | 1.00 |
| Only woman born in Australia | 2.14^**^ | 1.20 | 1.39 | 1.57^*^ | 0.98 |
| Total income (IHS) | 1.40^***^ | 1.30^***^ | 1.09 | 1.27^***^ | 0.90^*^ |
| Relationship history (ref. both 1^st^ relationship) |  |  |  |  |  |
| Men 1^st^ relationship and women 2^nd^+ | 0.22^***^ | 1.44 | 2.02^***^ | 1.67^*^ | 2.81^***^ |
| Women 1^st^ relationship and men 2^nd^+ | 0.32^***^ | 1.82^*^ | 1.63^*^ | 1.75^**^ | 2.32^***^ |
| Both 2^nd^+ relationship | 0.04^***^ | 5.16^***^ | 5.34^***^ | 9.81^***^ | 21.73^***^ |
| Relationship duration | 1.07^***^ | 0.98^*^ | 0.97^***^ | 0.95^***^ | 0.94^***^ |
| N (observations) | 15,263 | 15,263 | | | |
| N (couples) | 7,006 | 7,006 | | | |
| AIC/BIC | 11,084/11,229 | 40,707/41,264 | | | |

HILDA Survey (2002, 2006, 2010 & 2014). Odds ratios. All models feature robust standard errors. ^*^ *p<*0.05, ^**^ *p<*0.01, ^***^ *p<*0.001.
