# Supplementary material for: To pool or not to pool? Trends and predictors of banking arrangements within Australian couples
Source: PLoS One. 2019 Apr 17;14(4):e0214019. doi: 10.1371/journal.pone.0214019 (PMC6469846; doi:10.1371/journal.pone.0214019)
Supplement: S5 Table — HILDA Survey (2002, 2006, 2010 & 2014). Odds ratios. All models feature robust standard errors. * p<0.05, ** p<0.01, *** p<0.001. (DOCX) [file pone.0214019.s005.docx]

**Table S5. Banking arrangements among heterosexual couples in Australia, full output for models testing Hypothesis 4.**

|  | Joint account  vs. no joint  account | Banking arrangements (ref. partners have only a joint account) | | | |
| --- | --- | --- | --- | --- | --- |
|  |  | Joint+man separate | Joint+woman  separate | Joint+both  separate | Both separate only |
| Couple’s mean age | 1.06^***^ | 0.98^***^ | 0.99 | 0.98^***^ | 0.96^***^ |
| Couples’ age difference (<=5 years) |  |  |  |  |  |
| Man 5 years older | 0.46^***^ | 1.44^*^ | 1.13 | 1.42^*^ | 1.75^***^ |
| Woman 5 years older | 0.26^***^ | 1.39 | 1.73 | 2.32^**^ | 3.29^***^ |
| Marital status (*de facto*) |  |  |  |  |  |
| Married | 62.16^***^ | 0.10^***^ | 0.14^***^ | 0.04^***^ | 0.02^***^ |
| Employment status (neither employed) |  |  |  |  |  |
| Both employed | 3.22^***^ | 1.23 | 1.73^***^ | 1.23 | 0.62^**^ |
| Only man employed | 2.49^***^ | 1.54^*^ | 1.78^***^ | 1.11 | 0.80 |
| Only woman employed | 1.08 | 1.54 | 2.12^**^ | 1.77^*^ | 1.67^*^ |
| University degree (neither has degree) |  |  |  |  |  |
| Both have degrees | 1.43 | 1.93^***^ | 1.22 | 1.63^**^ | 1.36 |
| Only man has a degree | 1.16 | 1.52^*^ | 1.20 | 1.60^**^ | 1.30 |
| Only woman has a degree | 1.18 | 1.35 | 0.85 | 1.11 | 1.02 |
| Born in Australia (neither) ^a^ |  |  |  |  |  |
| Both born in Australia | 1.50^*^ | 1.22 | 1.65^***^ | 1.65^**^ | 1.22 |
| Only man born in Australia | 1.79^*^ | 1.96^**^ | 2.04^**^ | 2.47^***^ | 1.50 |
| Only woman born in Australia | 1.78^*^ | 1.40 | 1.56^*^ | 1.69^*^ | 1.12 |
| Total income (IHS) | 1.29^**^ | 1.35^***^ | 1.10 | 1.26^**^ | 0.95 |
| Gender-role attitudes | 1.00 | 1.00 | 0.99 | 0.99^***^ | 0.99^*^ |
| N (observations) | 14,081 | 14,081 | | | |
| N (couples) | 6,489 | 6,489 | | | |
| AIC/BIC | 10,014/10,142 | 37,726/38,217 | | | |

HILDA Survey (2002, 2006, 2010 & 2014). Odds ratios. All models feature robust standard errors. ^*^ *p<*0.05, ^**^ *p<*0.01, ^***^ *p<*0.001.
