# Supplementary material for: To pool or not to pool? Trends and predictors of banking arrangements within Australian couples
Source: PLoS One. 2019 Apr 17;14(4):e0214019. doi: 10.1371/journal.pone.0214019 (PMC6469846; doi:10.1371/journal.pone.0214019)
Supplement: S6 Table — HILDA Survey (2002, 2006, 2010 & 2014). Odds ratios. All models feature robust standard errors. * p<0.05, ** p<0.01, *** p<0.001. (DOCX) [file pone.0214019.s006.docx]

**Table S6. Banking arrangements among heterosexual couples in Australia, full output for models testing Hypothesis 5.**

|  | Joint account  vs. no joint  account | Banking arrangements (ref. partners have only a joint account) | | | |
| --- | --- | --- | --- | --- | --- |
|  |  | Joint+man separate | Joint+woman  separate | Joint+both  separate | Both separate only |
| Couple’s mean age | 1.06^***^ | 0.97^***^ | 0.99^*^ | 0.98^***^ | 0.96^***^ |
| Couples’ age difference (<=5 years) |  |  |  |  |  |
| Man 5 years older | 0.43^***^ | 1.44^*^ | 1.17 | 1.47^**^ | 1.86^***^ |
| Woman 5 years older | 0.31^***^ | 1.45 | 1.75 | 2.44^**^ | 3.14^***^ |
| Marital status (*de facto*) |  |  |  |  |  |
| Married | 63.40^***^ | 0.11^***^ | 0.15^***^ | 0.04^***^ | 0.02^***^ |
| Employment status (neither employed) |  |  |  |  |  |
| Both employed | 3.11^***^ | 1.20 | 1.76^***^ | 1.26 | 0.64^**^ |
| Only man employed | 2.46^***^ | 1.58^*^ | 1.71^***^ | 1.09 | 0.79 |
| Only woman employed | 1.09 | 1.68 | 2.25^***^ | 1.88^**^ | 1.71^*^ |
| University degree (neither has degree) |  |  |  |  |  |
| Both have degrees | 1.42 | 1.77^***^ | 1.30 | 1.62^**^ | 1.40 |
| Only man has a degree | 1.28 | 1.37 | 1.15 | 1.45^*^ | 1.19 |
| Only woman has a degree | 1.18 | 1.27 | 0.92 | 1.10 | 1.03 |
| Born in Australia (neither)^a^ |  |  |  |  |  |
| Both born in Australia | 1.54^*^ | 1.16 | 1.62^***^ | 1.68^***^ | 1.17 |
| Only man born in Australia | 1.86^*^ | 1.83^**^ | 2.01^**^ | 2.56^***^ | 1.45 |
| Only woman born in Australia | 1.68^*^ | 1.29 | 1.54^*^ | 1.77^**^ | 1.12 |
| Total income (IHS) | 1.30^***^ | 1.28^**^ | 1.09 | 1.27^***^ | 0.94 |
| Mean parental socio-economic status | 1.00 | 1.01 | 1.00 | 1.01^*^ | 1.00 |
| Family background (ref. neither from female-empowered family) |  |  |  |  |  |
| Only man from female-empowered family | 0.66 | 1.31 | 1.28 | 1.28 | 1.44 |
| Only woman from female-empowered family | 0.81 | 1.48 | 1.49 | 1.39 | 1.48 |
| Both from female-empowered family | 0.56^*^ | 1.24 | 1.33 | 1.26 | 1.67^*^ |
| N (observations) | 15,366 | 15,366 | | | |
| N (couples) | 7,044 | 7,044 | | | |
| AIC/BIC | 11,145/11,305 | 40,926/41,544 | | | |

HILDA Survey (2002, 2006, 2010 & 2014). Odds ratios. All models feature robust standard errors. ^*^ *p<*0.05, ^**^ *p<*0.01, ^***^ *p<*0.001.
