# Supplementary material for: To pool or not to pool? Trends and predictors of banking arrangements within Australian couples
Source: PLoS One. 2019 Apr 17;14(4):e0214019. doi: 10.1371/journal.pone.0214019 (PMC6469846; doi:10.1371/journal.pone.0214019)
Supplement: S7 Table — HILDA Survey (2002, 2006, 2010 & 2014). Column 1: random-effect binary logit models. Columns 2–4: random-effect multinomial logit models. All models feature robust standard errors. a controls: marital status, age, employment, education and ethnicity. b controls: marital status, age, employment, education, ethnicity and total income (IHS). c controls: age, employment, education, ethnicity and total income. * p<0.05, ** p<0.01, *** p<0.001. Complete tables of model coefficients are available from the authors upon request. (DOCX) [file pone.0214019.s007.docx]

**Table S7. Banking arrangements among heterosexual couples in Australia, HILDA Survey top-up sample excluded.**

|  | Joint account  vs. no joint  account | Banking arrangements (ref. partners have only a joint account) | | | |
| --- | --- | --- | --- | --- | --- |
|  |  | Joint+man separate | Joint+woman  separate | Joint+both  separate | Both separate only |
| *Hypothesis 1 ^a^* |  |  |  |  |  |
| Total income (IHS) | 1.30^***^ | 1.33^***^ | 1.09 | 1.32^***^ | 0.95 |
| Relative resources (ref. similar contribution) |  |  |  |  |  |
| Women contribute 60%+ | 0.77 | 1.26 | 1.62^***^ | 1.43^**^ | 1.59^***^ |
| Men contribute 60%+ | 1.09 | 1.11 | 1.28^**^ | 1.01 | 1.06 |
| N (observations) | 14,291 | 14,291 | | | |
| N (couples) | 5,966 | 5,966 | | | |
| AIC/BIC | 10,173/10,309 | 37,925/38,448 | | | |
| *Hypothesis 2 ^b^* |  |  |  |  |  |
| Number of dependent children | 1.35^***^ | 0.88^**^ | 0.93 | 0.78^***^ | 0.76^***^ |
| N (observations) | 14,291 | 14,291 | | | |
| N (couples) | 5,966 | 5,966 | | | |
| AIC/BIC | 10,140/10,269 | 37,856/38,348 | | | |
| *Hypothesis 3 ^c^* |  |  |  |  |  |
| Relationship history (ref. both 1^st^ relationship) |  |  |  |  |  |
| Men 1^st^ relationship and women 2^nd^+ | 0.19^***^ | 1.62 | 2.20^***^ | 1.83^*^ | 3.12^***^ |
| Women 1^st^ relationship and men 2^nd^+ | 0.29^***^ | 1.93^**^ | 1.69^*^ | 1.83^**^ | 2.48^***^ |
| Both 2^nd^+ relationship | 0.04^***^ | 5.19^***^ | 5.35^***^ | 10.31^***^ | 22.64^***^ |
| Relationship duration | 1.07^***^ | 0.98 | 0.98^**^ | 0.96^***^ | 0.94^***^ |
| N (observations) | 14,186 | 14,186 | | | |
| N (couples) | 5,929 | 5,929 | | | |
| AIC/BIC | 10,102/10,245 | 37,719/38,270 | | | |
| *Hypothesis 4 ^b^* |  |  |  |  |  |
| Gender-role attitudes | 1.00 | 1.00 | 0.99 | 0.99^***^ | 0.99^*^ |
| N (observations) | 13,122 | 13,122 | | | |
| N (couples) | 5,530 | 5,530 | | | |
| AIC/BIC | 9,152/9,279 | 35,041/35,527 | | | |
| *Hypothesis 5 ^b^* |  |  |  |  |  |
| Mean parental socio-economic status | 1.00 | 1.01 | 1.00 | 1.01^*^ | 1.00 |
| Family background (ref. neither from female-empowered family) |  |  |  |  |  |
| Only man from female-empowered family | 0.55^*^ | 1.40 | 1.50^*^ | 1.45 | 1.87^**^ |
| Only woman from female-empowered family | 0.67 | 1.45 | 1.40 | 1.46 | 1.58 |
| Both from female-empowered family | 0.85 | 1.39 | 1.46 | 1.36 | 1.42 |
| N (observations) | 14,279 | 14,279 | | | |
| N (couples) | 5,957 | 5,957 | | | |
| AIC/BIC | 10,159/10,318 | 37,913/38,526 | | | |

HILDA Survey (2002, 2006, 2010 & 2014). Column 1: random-effect binary logit models. Columns 2-4: random-effect multinomial logit models. All models feature robust standard errors. ^a^ controls: marital status, age, employment, education and ethnicity. ^b^ controls: marital status, age, employment, education, ethnicity and total income (IHS). ^c^ controls: age, employment, education, ethnicity and total income. ^*^ *p<*0.05, ^**^ *p<*0.01, ^***^ *p<*0.001. Complete tables of model coefficients are available from the authors upon request.
