# Supplementary material for: To pool or not to pool? Trends and predictors of banking arrangements within Australian couples
Source: PLoS One. 2019 Apr 17;14(4):e0214019. doi: 10.1371/journal.pone.0214019 (PMC6469846; doi:10.1371/journal.pone.0214019)
Supplement: S8 Table — HILDA Survey (2002, 2006, 2010 & 2014). Column 1: random-effect binary logit models. Columns 2–4: random-effect multinomial logit models. All models feature robust standard errors. a controls: marital status, age, employment, education and ethnicity. b controls: marital status, age, employment, education, ethnicity and total income (IHS). c controls: age, employment, education, ethnicity and total income. * p<0.05, ** p<0.01, *** p<0.001. Complete tables of model coefficients are available from the authors upon request. (DOCX) [file pone.0214019.s008.docx]

**Table S8. Banking arrangements among heterosexual couples in Australia, models where couple-level mismatches in joint bank accounts are coded as ‘no joint account’.**

|  | Joint account  vs. no joint  account | Banking arrangements (ref. partners have only a joint account) | | | |
| --- | --- | --- | --- | --- | --- |
|  |  | Joint+man separate | Joint+woman  separate | Joint+both  separate | Both separate only |
| *Hypothesis 1 ^a^* |  |  |  |  |  |
| Total income (IHS) | 1.25^**^ | 1.24^*^ | 1.20 | 1.30^**^ | 0.99 |
| Relative resources (ref. similar contribution) |  |  |  |  |  |
| Women contribute 60%+ | 0.71^*^ | 1.36 | 1.39^*^ | 1.37^*^ | 1.60^***^ |
| Men contribute 60%+ | 1.02 | 1.09 | 1.15 | 0.95 | 1.04 |
| N (observations) | 13,675 | 13,675 | | | |
| N (couples) | 6,645 | 6,645 | | | |
| AIC/BIC | 11,350/11,485 | 33,567/34,086 | | | |
| *Hypothesis 2 ^b^* |  |  |  |  |  |
| Number of dependent children | 1.33^***^ | 0.83^**^ | 0.83^***^ | 0.73^***^ | 0.73^***^ |
| N (observations) | 13,675 | 13,675 | | | |
| N (couples) | 6,645 | 6,645 | | | |
| AIC/BIC | 11,323/11,451 | 33,513/34,002 | | | |
| *Hypothesis 3 ^c^* |  |  |  |  |  |
| Relationship history (ref. both 1^st^ relationship) |  |  |  |  |  |
| Men 1^st^ relationship and women 2^nd^+ | 0.18^***^ | 1.48 | 2.52^***^ | 2.02^**^ | 3.29^***^ |
| Women 1^st^ relationship and men 2^nd^+ | 0.27^***^ | 1.99^*^ | 2.56^***^ | 2.22^***^ | 3.01^***^ |
| Both 2^nd^+ relationship | 0.03^***^ | 6.82^***^ | 8.78^***^ | 15.10^***^ | 33.49^***^ |
| Relationship duration | 1.07^***^ | 0.96^***^ | 0.95^***^ | 0.94^***^ | 0.92^***^ |
| N (observations) | 13,565 | 13,565 | | | |
| N (couples) | 6,600 | 6,600 | | | |
| AIC/BIC | 11,306/11,449 | 33,326/33,874 | | | |
| *Hypothesis 4 ^b^* |  |  |  |  |  |
| Gender-role attitudes | 1.01 | 1.01 | 0.99 | 0.99^***^ | 0.99^**^ |
| N (observations) | 12,464 | 12,464 | | | |
| N (couples) | 6,089 | 6,089 | | | |
| AIC/BIC | 10,232/10,358 | 30,840/31,323 | | | |
| *Hypothesis 5 ^b^* |  |  |  |  |  |
| Mean parental socio-economic status | 1.00 | 1.01 | 1.01 | 1.01^**^ | 1.01 |
| Family background (ref. neither from female-empowered family) |  |  |  |  |  |
| Only man from female-empowered family | 0.49^**^ | 1.34 | 1.34 | 1.36 | 1.77^**^ |
| Only woman from female-empowered family | 0.55^*^ | 1.29 | 1.32 | 1.38 | 1.55 |
| Both from female-empowered family | 0.76 | 1.72^*^ | 1.59 | 1.56 | 1.61 |
| N (observations) | 13,663 | 13,663 | | | |
| N (couples) | 6,636 | 6,636 | | | |
| AIC/BIC | 11,329/11,487 | 33,549/34,158 | | | |

HILDA Survey (2002, 2006, 2010 & 2014). Column 1: random-effect binary logit models. Columns 2-4: random-effect multinomial logit models. All models feature robust standard errors. ^a^ controls: marital status, age, employment, education and ethnicity. ^b^ controls: marital status, age, employment, education, ethnicity and total income (IHS). ^c^ controls: age, employment, education, ethnicity and total income. ^*^ *p<*0.05, ^**^ *p<*0.01, ^***^ *p<*0.001. Complete tables of model coefficients are available from the authors upon request.
