# Supplementary material for: To pool or not to pool? Trends and predictors of banking arrangements within Australian couples
Source: PLoS One. 2019 Apr 17;14(4):e0214019. doi: 10.1371/journal.pone.0214019 (PMC6469846; doi:10.1371/journal.pone.0214019)
Supplement: S9 Table — HILDA Survey (2002, 2006, 2010 & 2014). Column 1: fixed-effect binary logit models. Columns 2–4: fixed-effect multinomial logit models. Couples’ age difference and born in Australia are time constant and dropped in the models. (DOCX) [file pone.0214019.s009.docx]

**Table S9. Banking arrangements among heterosexual couples in Australia, fixed-effect specifications.**

|  | Joint account  vs. no joint  account | Banking arrangements (ref. partners have only a joint account) | | | |
| --- | --- | --- | --- | --- | --- |
|  |  | Joint+man separate | Joint+woman  separate | Joint+both  separate | Both separate only |
| *Hypothesis 1* |  |  |  |  |  |
| Total income (IHS) | 1.03 | 1.26 | 1.07 | 1.12 | 1.10 |
| Relative resources (ref. similar contribution) |  |  |  |  |  |
| Women contribute 60%+ | 0.93 | 1.03 | 1.33 | 1.05 | 1.16 |
| Men contribute 60%+ | 1.26 | 0.99 | 1.07 | 0.92 | 0.75 |
| N(observations) | 2,114 | 7,253 | | | |
| N(individuals) | 699 | 2,199 | | | |
| AIC/BIC | 1,305/1,367 | 5,758/6,061 | | | |
| *Hypothesis 2* |  |  |  |  |  |
| Number of dependent children | 1.43^***^ | 0.83^*^ | 0.96 | 0.73^***^ | 0.56^***^ |
| N(observations) | 2,114 | 7,253 | | | |
| N(individuals) | 699 | 2,199 | | | |
| AIC/BIC | 1,284/1,341 | 5,710/5,986 | | | |
| *Hypothesis 4* |  |  |  |  |  |
| Gender-role attitudes | 1.00 | 1.00 | 1.00 | 0.99 | 1.00 |
| N(observations) | 1,847 | 6,627 | | | |
| N(individuals) | 614 | 2,027 | | | |
| AIC/BIC | 1,160/1,215 | 5,265/5,537 | | | |

HILDA Survey (2002, 2006, 2010 & 2014). Column 1: fixed-effect binary logit models. Columns 2-4: fixed-effect multinomial logit models. Couples’ age difference and born in Australia are time constant and dropped in the models.
